# BepiColombo cruise science: overview of the mission contribution to heliophysics

**DOI:** 10.1186/s40623-025-02256-z

**Published:** 2025-07-17

**Authors:** Beatriz Sánchez-Cano, Lina Z. Hadid, Sae Aizawa, Go Murakami, Yumi Bamba, Shota Chiba, Takuya Hara, Daniel Heyner, George Ho, Kazumasa Iwai, Emilia Kilpua, Gaku Kinoshita, Benoit Lavraud, Yoshizumi Miyoshi, Marco Pinto, Daniel Schmid, Daikou Shiota, Rami Vainio, Nicolas Andre, Alessandro Aronica, Sami Asmar, Hans-Ulrich Auster, Stas Barabash, Alain Barthe, Wolfgang Baumjohann, Johannes Benkhoff, Mark Bentley, Emma Bunce, Paolo Cappuccio, Dominique Delcourt, Ivan di Stefano, Irene Doria, Nina Dresing, Andrei Fedorov, David Fischer, Bjorn Fiethe, Markus Fränz, Jan Gieseler, Franz Giner, Gabriel Giono, Yuki Harada, Hauke Hussmann, Luciano Iess, Takeshi Imamura, Harald Jeszenszky, Geraint Jones, Bruno Katra, Adrian Kazakov, Alexander Kozyrev, Gunter Laky, Carlo Lefevre, Herbert Lichtenegger, Simon Lindsay, Marco Lucente, Carmelo Magnafico, Werner Magnes, Adrian Martindale, Ayako Matsuoka, Anna Milillo, Igor Mitrofanov, Gaku Nishiyama, Philipp Oleynik, Stefano Orsini, Meegyeong Paik, Christian Palmroos, Christina Plainaki, Emanuel Penou, Moa Persson, Francesco Quarati, Eric Quémerais, Ingo Richter, Rozenn Robidel, Mathias Rojo, Yoshifumi Saito, Francesco Santoli, Alexander Stark, Mirko Stumpo, Rong Tian, Ali Varsani, Christopher Verdeil, Hayley Williamson, Olivier Witasse, Shoichiro Yokota

**Affiliations:** 1 School of Physics and Astronomy, University of Leicester, Leicester, UK; 2 Laboratoire de Physique des Plasmas (LPP), CNRS, École Polytechnique, Institut Polytechnique de Paris, Observatoire de Paris, Sorbonne Université, Université Paris Saclay, Palaiseau, France; 3 Japan Aerospace Exploration Agency (JAXA), Tokyo, Japan; 4 National Institute of Information and Communication Technology, Tokyo, Japan; 5 Institute for Space-Earth Environmental Research, Nagoya University, Furo-cho, Chikusa, Nagoya, Aichi 464-8601 Japan; 6 Graduate School of Frontier Sciences, The University of Tokyo, Tokyo, Japan; 7 Space Sciences Laboratory, University of California, Berkeley, CA USA; 8 Institut für Geophysik und extraterrestrische Physik, Technische Universität Braunschweig, Brunswick, Germany; 9 Southwest Research Institute, San Antonio, TX USA; 10 Department of Physics, University of Helsinki, Helsinki, Finland; 11 Department of Earth and Planetary Science, The University of Tokyo, Tokyo, Japan; 12 Laboratoire d’astrophysique de Bordeaux, CNRS, University of Bordeaux, Pessac, France; 13 European Space Research and Technology Centre (ESTEC), European Space Agency, Noordwijk, Netherlands; 14 Space Research Institute, Austrian Academy of Sciences, Graz, Austria; 15 Department of Physics and Astronomy, University of Turku, Turku, Finland; 16 Institut de Recherche en Astrophysique et Planétologie (IRAP), CNRS, CNES, Université Toulouse III, Toulouse, France; 17 Institut Supérieur de l’Aéronautique et de l’Espace (ISAE‐SUPAERO), Université de Toulouse, Toulouse, France; 18 NASA’s Jet Propulsion Laboratory, California Institute of Technology, Pasadena, CA USA; 19 European Space Astronomy Centre (ESAC), European Space Agency, Villafranca del Castillo, Villanueva de la Cañada, Madrid, Spain; 20 Department of Mechanical and Space Engineering (DIMA), Sapienza University of Rome, Via Eudossiana 18, 00184 Rome, Italy; 21 IDA, Brunswick, Germany; 22 Max Planck Institute for Solar System Research (MPS), Göttingen, Germany; 23 Kyoto University, Kyoto, Japan; 24 Space Research Institute of the Russian Academy of Sciences (IKI), Moscow, Russia; 25 World Data Center for Geomagnetism, Graduate School of Science, Kyoto University, Kyoto, Japan; 26 INAF-Istituto di Astrofisica e Planetologia Spaziali, 00133 Rome, Italy; 27 Swedish Institute of Space Physics, Uppsala, Sweden; 28 LATMOS-OVSQ, Université Versailles Saint-Quentin, Guyancourt, France; 29 Swedish Institute of Space Physics, Kiruna, Sweden; 30 Osaka University, Osaka, Japan; 31 Agenzia Spaziale Italiana (ASI), Frascati, Italy; 32 Department of Radiation Science and Technology, Faculty of Applied Sciences, Delft University of Technology, Mekelweg 15, 2629 JB Delft, Netherlands; 33 Gonitec BV, Johannes Bildersstraat 43, 2596 EE Den Haag, The Netherlands; 34 German Aerospace Center (DLR), Institute of Space Research, Berlin, Germany

**Keywords:** BepiColombo, Cruise phase, Solar wind, Solar energetic particles, Coronal mass ejections

## Abstract

BepiColombo, the joint ESA/JAXA mission to Mercury, was launched in October 2018 and is scheduled to arrive at Mercury in November 2026 after an 8-year cruise. Like other planetary missions, its scientific objectives focus mostly on the nominal, orbiting phase of the mission. However, due to the long duration of the cruise phase covering distances between 1.2 and 0.3 AU, the BepiColombo mission has been able to outstandingly contribute to characterise the solar wind and transient events encountered by the spacecraft, as well as planetary environments during the flybys of Earth, Venus, and Mercury, and contribute to the characterisation of the space radiation environment in the inner Solar System and its evolution with solar activity. In this paper, we provide an overview of the cruise observations of BepiColombo, highlighting the most relevant science cases, with the aim of demonstrating the importance of planetary missions to perform cruise observations, to contribute to a broader understanding of Space Weather in the Solar System, and in turn, increase the scientific return of the mission.

**Graphical Abstract:**

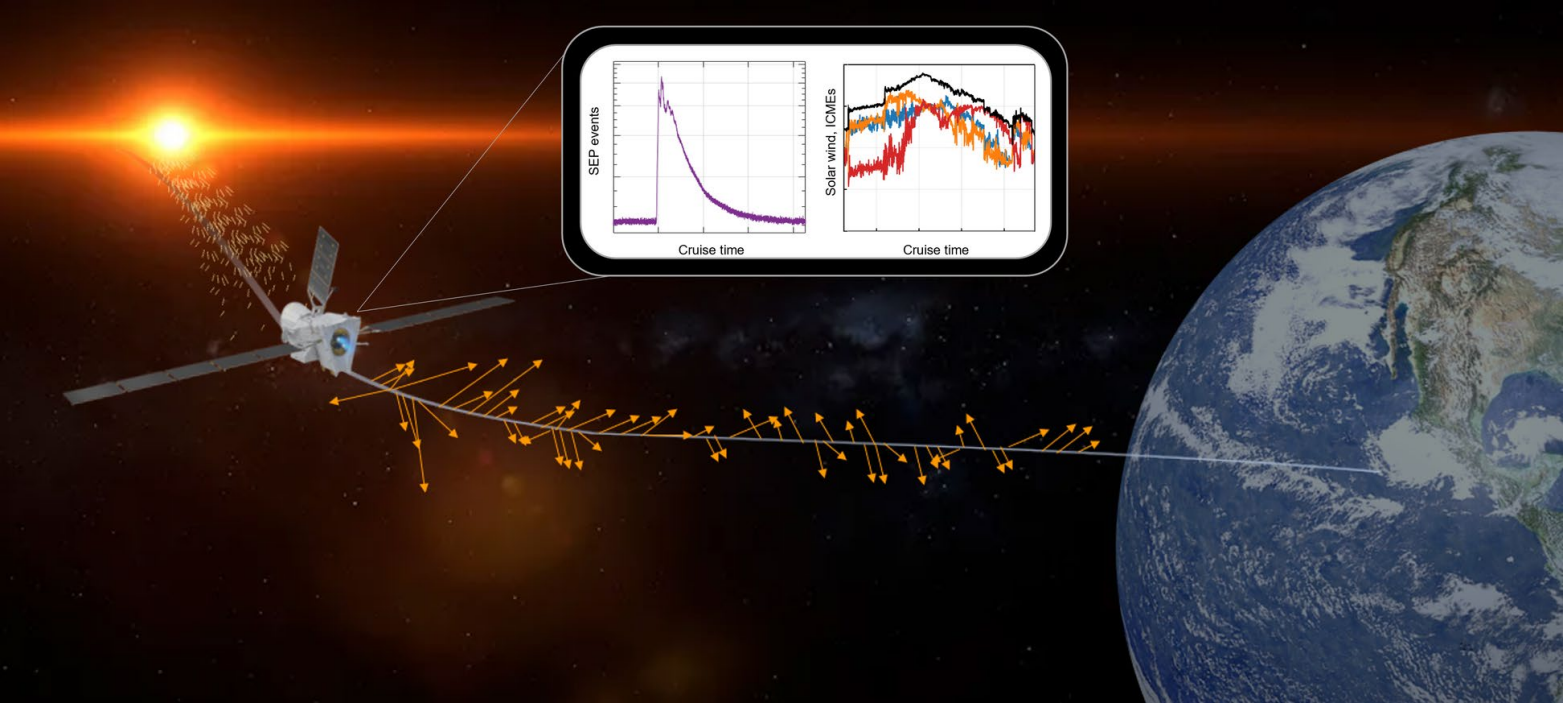

## Introduction: the BepiColombo cruise science goals

In the new era of planetary and heliophysics/solar missions transiting the very inner heliosphere, such as BepiColombo (Benkhoff et al. 2021), Solar Orbiter (Müller et al. 2020), Parker Solar Probe (Fox et al. 2016) or the Jupiter Icy Moons Explorer (JUICE, Grasset et al. 2013), a new and unique opportunity to perform combined multi-point observations of the interplanetary medium is presented, which is of enhanced interest when combined with other assets at different distances, such as at Venus (0.7 AU), at 1 AU (i.e. Earth, the Solar TErrestrial RElations Observatory, STEREO-A, Kaiser et al. 2008), or even at further distances such as Mars (1.38–1.66 AU) and Jupiter (5.2 AU). The varying radial and longitudinal separations between BepiColombo and other current heliospheric missions provide excellent and unique opportunities to study the structure and evolution of Interplanetary Coronal Mass Ejections (ICME) and slow-fast Stream Interactions Regions (SIRs) in the inner Solar System as we know they drive strong dynamics in the solar wind and have an effect on the propagation of Solar Energetic Particle (SEP) events.

In recent years, coordinated observations among different missions have allowed us to perform valuable investigations of the heliosphere from different points of view and address many aspects of plasma processes related to the Sun and solar wind interactions with planetary environments (Hadid et al. 2021). Moreover, several planetary missions in their way to their final target have been performing observations during their cruises such as the Rosetta mission (Taylor et al. 2017), or the Mercury Surface, Space Environment, Geochemistry and Ranging (MESSENGER, Solomon et al. 2007) mission. These missions provided additional opportunities for synergistic data acquisitions from environments and conditions that are different from each mission’s original baseline science operation plan. One of these missions is BepiColombo, which is a joint collaborative space mission between the European Space Agency (ESA) and the Japan Aerospace Exploration Agency (JAXA) designed for the characterisation of Mercury, including its composition, surface processes, geophysics, atmosphere, magnetosphere, and interaction with the solar wind and Space Weather (Benkhoff et al. 2021). BepiColombo is composed of two spacecraft. The European module is the Mercury Planetary Orbiter (MPO) that is planned to orbit between 480 and 1500 km above the surface of Mercury (Benkhoff et al. 2021), and the Mercury Magnetospheric Orbiter (MMO), also called *Mio*, developed and built in Japan (Murakami et al. 2020), planned to orbit between 590 and 11,640 km above the surface of the planet.

**Fig. 1:**
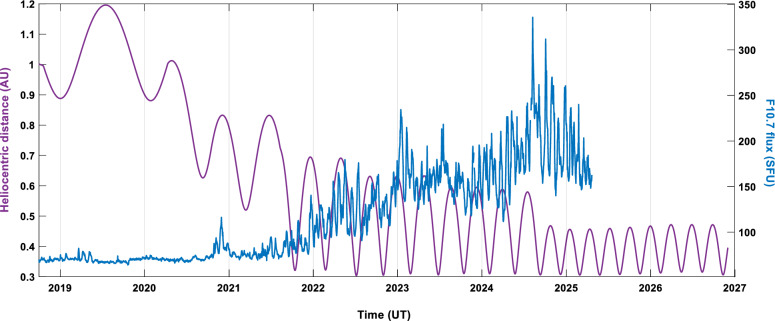
Heliocentric distance (purple) and solar flux via adjusted F10.7 cm proxy (blue, until the time of writing) covered by BepiColombo during the cruise phase.

BepiColombo was launched on 20 October 2018 from the Guiana Space Centre, and it is planned to be inserted in orbit around Mercury in November 2026, after a near 8-year cruise, including one flyby of Earth, two of Venus, and six flybys of Mercury (Mangano et al. 2021). During the cruise phase, both modules of the mission are travelling together in a packed configuration, with a common electric propulsion module named the Mercury Transfer Module (MTM) and a sun-shield named the Magnetospheric Orbiter Sunshield and Interface Structure (MOSIF). During this phase, several instruments are routinely in operation, such as the magnetometer (MPO-MAG, Heyner et al. 2021), the Solar Intensity X-ray and particle Spectrometer (SIXS, Huovelin et al. 2020), the BepiColombo Environment Radiation Monitor (BERM, Pinto et al. 2022) and the Mercurian Gamma-ray and Neutron Spectrometer (MGNS, Mitrofanov et al. 2021) of the MPO spacecraft. During the planetary flybys, as well as during calibration checks, most of the mission payloads that have their field-of-views unobstructed are also typically operated (Montagnon et al. 2021). Moreover, when particular configurations with other spacecraft are of scientific interest for the community, several other instruments can operate for short periods of time (Hadid et al. 2021). Therefore, the long journey of BepiColombo in the inner Solar System brings us the opportunity to contribute to the analysis of long-term variability in the solar wind, mainly Interplanetary Magnetic Fields (IMF), solar transient events, and Galactic Cosmic Rays (GCRs). As seen in Fig. 1, the trip covers half of a solar cycle, starting at the solar minimum of solar cycles 24–25, and reaching Mercury during the maximum solar activity of solar cycle 25, covering heliocentric distances between 1.2 and 0.3 AU. The unique orbit of this journey, together with Solar Orbiter, Parker Solar Probe, and other available space missions, is an important asset for heliophysics/solar science.

Among the scientific objectives of BepiColombo during the cruise phase, in coordination with another spacecraft in the inner heliosphere, are:

1. Investigations of solar wind processes (e.g., turbulence, waves, e.g., Telloni et al. 2022), structures (e.g., magnetic holes, e.g., Volwerk et al. 2023) or transient events (e.g., SEPs e.g., Dresing et al. 2023, ICMEs e.g., Palmerio et al. 2024a) and their evolution with heliocentric distance and solar activity.
2. Contributing to characterise planetary radiation environments (i.e. Space Weather) encountered during the flybys of Earth, Venus, and Mercury (e.g., Persson et al. 2022; Aizawa et al. 2022; Harada et al. 2022; Orsini et al. 2022; Hadid et al. 2024; Rojo et al. 2024).
3. Acting as upstream solar wind monitor to planets such as Venus or Mars (e.g., Khoo et al. 2024; Chi et al. 2023).
4. Characterising the evolution of GCR fluxes with the solar cycle and at heliocentric distances below 1 AU (e.g., Pinto et al. 2022), where not many observations of this kind are available.
5. Calibration of instruments with solar wind and flyby observations, as well as with respect to other satellites when they are nearby (e.g., Khoo et al. 2024; Rojo et al. 2023).

The main goal of this paper is to provide an overview of the science achieved by the BepiColombo mission during its cruise phase, and to demonstrate how cruise observations are a unique opportunity to significantly contribute to both the planetary and heliophysics communities by characterising Space Weather in the inner Solar System. This work is the result of a large international effort between the BepiColombo payload teams, ESA and JAXA, collaboration with other solar and planetary missions, and with the support of the Institute for Space-Earth Environmental (ISEE) Research of Nagoya University in Japan.

## BepiColombo instruments in operation during the cruise phase

**Fig. 2:**
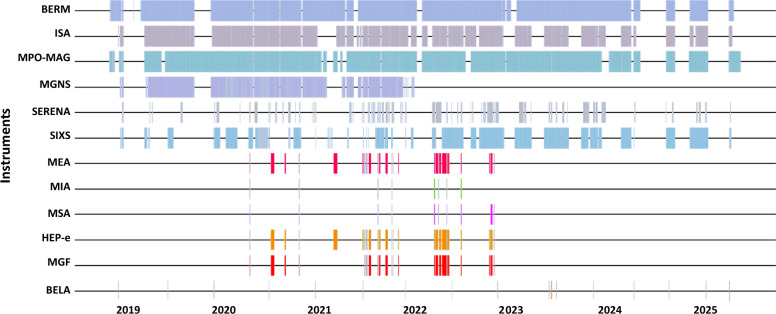
In situ instrument coverage based on product meta-data archived at the ESA Planetary Science Archive. The interactive version of this plot can be accessed at https://www.cosmos.esa.int/web/bepicolombo-yssg-cs/data-coverage

This section describes the BepiColombo in situ and remote instruments that can take observations during the cruise and the type of observations they get. We note that the mission has other instruments that will only be in operation during the flybys or in orbit about Mercury, this is why they are not described in this paper. Figure 2 shows the global coverage of in situ instruments up to the time of writing based on product meta-data archived at the ESA Planetary Science Archive.

*BELA*: The BepiColombo Laser Altimeter (BELA) is the first European laser altimeter for a planetary mission. It will measure ranges between the MPO spacecraft and the Mercury surface to observe the global topography, tidal deformation and rotation state of Mercury (Thomas et al. 2022). To facilitate these geodetic observations, BELA actively transmits a laser pulse at the wavelength of 1064 nm and receives the return pulse from the Mercury surface to measure its time-of-flight between MPO and Mercury. For each range measurement, performed ten times per second, BELA digitises the shape of the return pulse with a time resolution of 12.5 ns. However, such observations can be also conducted in passive mode, where no laser pulse was emitted. In such case, the detected signal is caused by energetic particles hitting the instrument. While the field-of-view of BELA’s telescope has been fully blocked by the MTM during the cruise phase, energetic particles that are capable of penetrating the spacecraft and depositing sufficient energy in BELA’s detector may generate signals with amplitudes exceeding the dark noise level. The detector is an infrared enhanced Silicon Avalanche Photodiode (Si APD) with an 812-µm-diameter circular sensitive area. During the cruise phase, BELA has been sporadically operated in passive mode, including two of the six Mercury flybys, in order to detect events caused by energetic particles, such as GCRs.

*BERM*: The BepiColombo Environment Radiation is a platform instrument aboard the Mercury Planetary Orbiter (MPO) that measures energetic particles, mainly SEP electrons and ions, and GCRs (Pinto et al. 2022). It consists of a single silicon stack telescope with a particle entrance of 0.5 mm^2^ and a 50-µm-thick beryllium cutoff window. Particles are separated into five channels for electrons (0.15–10 MeV), eight channels for protons (1.5–100 MeV), and five channels for heavy ions (1–50 MeV mg^−1^ cm^−2^). BERM is placed in the radiation panel of the MPO facing the anti-sunward direction. BERM is in operation during the whole cruise phase, including solar electric propulsions periods (since 2021), and it is only disconnected under specific operational circumstances.

*ISA*: The Italian Spring Accelerometer (ISA) is an accelerometer that measures the so-called non-gravitational perturbations, i.e. the accelerations perturbing the free fall trajectory of the spacecraft (Santoli et al. 2020). Although ISA’s main science goal is not heliophysics, it has significantly contributed to the understanding of the spacecraft behaviour in the solar wind. The instrument has been in background science operations for a relevant part of the cruise, covering special operations during all flybys (excluding the first Venus and the fifth Mercury flybys). Despite most of the data are used for internal calibration and performance monitoring, ISA had occasion to be the first accelerometer to directly measure the tidal effects (gravity gradients) of an extraterrestrial body on a spacecraft (Magnafico et al. 2025). The Venus flyby 2 was of particular interest for cruise science operations as it recorded a very large, spurious, signature in the acceleration readings lasting a few minutes during the closest approach. The acceleration peak was isolated and, using an estimation technique involving the applied on-board reaction wheels controlling torques, was revealed as the action of a superficial force applied on the MPO radiator side. The navigation estimations also confirmed the amplitude of the delta velocity affected the MCS (Magnafico et al. 2025). Further analysis on the MPO radiator temperatures, MCS attitude with respect to planet Venus and the abundance of water revealed by the other instruments, thus allowing the discovery of a major outgassing event on the spacecraft (De Filippis et al. 2025). During the Earth and some of Mercury flybys, BepiColombo has been also exposed to eclipse passages. ISA recorded the Solar Radiation Pressure variations effects on BepiColombo trajectory, which was used as unmodeled dynamical perturbations in the first combined data analysis of BepiColombo and MErcury Surface, Space ENvironment, GEochemistry, and Ranging (MESSENGER) radiometric measurements (Del VecChio et al. 2024).

*MERMAG (MPO-MAG and MGF)*: MERMAG (abbreviation of “Mercury magnetic field”) is a fluxgate magnetometer suite aboard the BepiColombo mission. Magnetometer onboard the MPO spacecraft instrument of BepiColombo (MPO-MAG, Heyner et al. 2021) studies the planetary magnetic field such as dynamo-generated field, crustal field, induction field, and external field generated by the magnetospheric currents at a sampling rate of 128 Hz. The magnetometer onboard the Mio spacecraft (MGF instrument, whereby MGF stands for magnetic field, Baumjohann et al. 2020) studies the plasma and magnetic field dynamics in the Mercury magnetosphere and inner heliosphere, also on sampling rates of up to 128 Hz. Once in a stable orbit around the planet, the two magnetometers will provide unprecedented, simultaneous two-point magnetic field measurements of Mercury’s space environment. During cruise phase the mast on Mio is stowed and MGF is only switched on occasionally (see Fig. 2), unlike the MPO magnetometer boom that was deployed right after launch. This gives the unique opportunity to measure the solar wind characteristics between Earth and Mercury over a long period of time. During the cruise phase, MPO-MAG is in operation all the time including solar electric propulsions periods (since 2021), but it has to be noted that the quality of the IMF observations is compromised during the propulsion periods and a cleaning analysis must be done before any scientific analysis, as done in Palmerio et al. (2024a). MPO-MAG is only switched off under specific operational circumstances. MGF is in operation only during specific solar wind or flyby campaigns.

*MGNS*: The Mercurian Gamma-ray and Neutron Spectrometer MGNS (Mitrofanov et al. 2021) is one of the payloads that are fully operative during the cruise phase (excluding solar electric propulsions periods). The MGNS spectrometers measure neutron fluxes from thermal energy up to 10 MeV and gamma-rays in the energy range of 300 keV up to 10 MeV with energy resolution of 5% at 662 keV and of 2% at 8 MeV, but also are sensitive to SEP events and GCRs that impact the spacecraft and are typically seen in their background counts. However, it is important to note that during the cruise phase, these measurements are impacted by the surrounding material of the composite stack (Mangano et al. 2021).

*MIXS*: The Mercury Imaging X-ray Spectrometer (MIXS, Bunce et al. 2020) is a payload of the MPO orbiter designed to map Mercury’s surface elemental composition by observing fluorescent X-rays generated when solar X-rays and/or energetic particles interact with the planet’s surface. In addition, it will determine the dynamic interaction of the planet’s surface with the surrounding space environment. It is composed of MIXS-C, which is a collimated instrument that will provide the global coverage at a spatial resolution of 50–100 km, and MIXS-T, which is an X-ray telescope that will reveal the X-ray flux from Mercury at better than 10 km resolution. The instrument uses DEPFET based detectors (Majewski et al. 2012) to achieve high spatial, energy and time resolution. MIXS cannot operate in its nominal science configuration during the cruise phase or flybys as its FOV is fully covered by the MTM in cruise configuration. However, it became apparent during commissioning and early cruise checkout operations that the instrument is sensitive to GCRs which are able to penetrate the spacecraft structure and deposit energy within the detector substrate either directly or (more probably) through the generation of particle showers in the spacecraft structure. In order to characterise this background signal for the science phase and act as a GCR counter in the inner heliosphere, MIXS has been operated sporadically throughout the cruise phase, as well as during five of the six Mercury planetary flybys.

*MORE*: MORE (Mercury Orbiter Radio science Experiment), the dedicated radio science component of the BepiColombo mission (Iess et al. 2021), will utilise an advanced radio tracking system to study Mercury’s gravitational field and its rotational state, to perform test of general relativity and alternative gravitational theories and to probe the solar corona. The key onboard elements are the deep space transponder (DST) and the Ka-band transponder (KaT): the DST manages an X-band uplink at 7.2 GHz, coherently paired with both an X-band downlink at 8.4 GHz and a Ka-band downlink at 32.0 GHz, whereas the KaT enables a coherent Ka/Ka link (uplink at 34.4 GHz, downlink at 32.1 GHz). All carriers are phase-modulated with a standard pseudo-noise (PN) ranging signal at a chip rate of approximately 3 Mcps (Megachips per second). Additionally, the KaT features an innovative wideband ranging system (WBRS) exploiting a custom PN ranging implementation operating at 24.3 Mcps (Ciarcia et al. 2013), able to provide accurate ranging measurements. The primary data used for scientific investigations are the Ka/Ka measurements provided by the KaT, while the X/X and X/Ka measurements are used to calibrate the dispersive noise caused by solar plasma, especially during superior solar conjunctions. This multi-frequency calibration scheme proved to be effective at almost all solar elongation angles. ESA’s DSA3 antenna (Malargüe, Argentina) serves as the main ground station for the MORE experiment. This antenna supports multi-frequency links and the novel WBRS employed by MORE. It is endowed with a water vapour radiometer known as Tropospheric Delay Calibration System (TDCS), to calibrate for the noise induced by Earth’s troposphere (Lasagni-Manghi et al. 2022). DSA3 is also equipped with an online delay calibration system for Ka-band data correcting for signal delays caused by ground station operational equipment and reflective mirrors. Additional ground support for MORE will be provided by NASA’s DSS-25 antenna at the Goldstone complex in California. DSS-25 successfully showcased its ability to support MORE’s WBRS during a 2019 experimental campaign (Cappuccio et al. 2020). During the cruise, MORE is in operation only during specific campaigns.

*MPPE (MSA, MIA, MEA, HEP)*: Mercury Plasma Particle Experiments (MPPE) is a consortium onboard Mio spacecraft of the BepiColombo mission developed at JAXA to explore Mercury’s plasma environment. It consists of seven sensors to cover both ions and electrons from low (a few eV) to high (a few MeV) energy (Saito et al. 2010, 2021). During the cruise phase, BepiColombo is in the stacked configuration and the MPPE consortium is placed inside the sunshield. Thus, most of the observation window is blocked and the solar wind protons cannot be observed under quiet solar wind conditions. Despite its limited field-of-view of the instruments, some of the MPPE sensors are occasionally turned on and can measure electrons and ions in particular when solar events occur, such as CMEs or SEPs. Cruise observations by the MPPE are mainly made by the Mercury Electron Analyzer (MEA) which observes low energy electrons with a few eV up to a few keV in the solar wind, the Mercury Ion Analyzer (MIA) which operates from a few eV/q up to 30 keV/q and the Mercury mass Spectrum Analyzer (MSA) which measures the ions from a few eV/q up to ~38 keV/q. MEA is the electrostatic electron analyzer and it is composed of two sensors (MEA1 and MEA2). During cruise observation, only MEA1 is switched on and conducts its observations. Different energy tables (3–300 eV, 3 eV–3 keV, 3 eV–26 keV) have been used at different periods of time. Incoming electrons are selected according to their energies by electrostatic deflection in symmetrical toroidal analyzers. Although the field-of-view is very limited, we are able to derive the density and temperature from cruise data and make them available (Rojo et al. 2023). While MIA is a toroidal top-hat electrostatic energy analyzer that measures ions without mass distinction, MSA is designed to provide three-dimensional mass-resolved ion phase space densities in Mercury’s magnetosphere (Delcourt et al. 2016). This ion spectrometer combines a spherical top-hat analyzer for energy analysis with a “Time-Of-Flight” (TOF) chamber for mass analysis. At the entrance of the TOF analyzer, the ions interact with carbon foils and as a result of charge exchange, may exit as neutrals, positive or negative ions. Unlike most spectrometers onboard spacecraft that feature an equipotential TOF chamber, a prominent characteristic of MSA is that the TOF chamber is linearly polarised which allows us to correct energy and angular scattering upon entry of the positive ions into the TOF chamber. This original “reflectron” concept (Managadze 1986) leads to isochronous TOF; hence an enhanced mass resolution (m/delam > 40). On the other hand, most of the incoming ions measured by MSA exit the carbon foils as neutrals or negative ions. These ions have “Straight Through” (ST) trajectories toward the bottom end of the MSA TOF chamber. Throughout the cruise phase, because of a limited telemetry rate, MPO and Mio data are produced in low resolution and only ST data produced by MSA are transmitted. The MPPE suite also has a couple of sensors to measure High Energy Particles (HEP), both electrons (HEP-e) and ions (HEP-i). During the cruise phase, HEP-e is in operation during some specific intervals of time as it has a radial field-of-view, but HEP-i is typically not in operation as the conical field-of-view is blocked by the spacecraft shield. HEP-e measures electrons in the range 30–700 keV and ions in the range 30 keV–1.5 MeV. The MPPE suite is in operation only during specific solar wind or flyby campaigns. One feature observed during cruise phase and planetary flybys by MPPE is a continuous outgassing of water from the spacecraft (Fränz et al. 2024).

*PHEBUS*: PHEBUS (standing for Probing the Hermean Exosphere By Ultraviolet Spectroscopy) is a double spectrometer (EUV and FUV detectors) with two additional visible channels (c404 and c422 channels, Quémerais et al. 2020). The EUV detector actually operates in the Extreme and Far UltraViolet (55–155 nm) while the FUV detector operates in the far, middle and near ultraviolet (145–315 nm). The c404 channel is centred at the emission line of potassium (404.7 nm) while the c422 channel is centred at the emission line of calcium (422.8 nm). The instrument is located on the radiator panel of MPO, with a rotating baffle extending outside the radiator panel. A combination of a rotating primary mirror and baffle allows changing the pointing direction of PHEBUS field-of-view, independently of the spacecraft attitude. When the instrument is not operating, the baffle is positioned in front of a parking bracket which blocks the light, and this is called the safe position. During the cruise, PHEBUS operates sporadically during stellar observation campaigns, interplanetary background observations and planetary flybys, and allows to infer SEP events.

*SERENA (MIPA and PICAM)*: SERENA (search for exospheric refilling and emitted natural abundances) is an experiment composed of four sensors of complementary neutral and ionised particle detectors on board MPO. During the cruise phase, BepiColombo is in the stacked configuration and only the ion sensors MIPA and PICAM are able to perform scientific observations. The Miniaturized Ion Precipitation Analyzer (MIPA) is one of the four sensors that form the Search for Exospheric Refilling and Emitted Natural Abundances (SERENA) suite (Orsini et al. 2021). MIPA is a small ion mass spectrometer with a hemispherical field-of-view optimised for the study of solar wind precipitation. MIPA has a configurable electrostatic deflection entrance system, giving a variable angular resolution ranging from 8° to 60°, followed by an electrostatic analyzer (ESA) capable of measuring ions with energies from 10 eV to 15 keV with a resolution of 7.3%. A full energy-angle scan takes 20 s. After passing through the ESA, ions pass into a time-of-flight (TOF) cell for mass analysis, where they scatter off a start surface such that secondary electrons are detected by a START channel electron multiplier (CEM), then impact on a stop surface, producing secondary electrons detected by the STOP CEM. The time between the START and STOP CEM detections is used to infer the particle mass with low resolution. While the CEMs have low background noise rates, penetrating high-energy particles from a SEP or CME can directly impact either CEM and produce artificial counts. The MIPA field-of-view is not blocked in the stacked configuration, so the sensor has been able to operate at full capacity sporadically throughout the cruise phase. The Planetary Ion CAMera (PICAM) is an ion mass spectrometer operating as an all-sky camera for ions. It is one of the four sensors that form the SERENA suite on BepiColombo’s MPO. PICAM studies the exospheric ions around Mercury, and together with the other SERENA sensors aims at understanding the chain of processes by which neutrals are ejected from the surface of Mercury, ionised and transported through the environment surrounding the planet. The design of its electrostatic analyser facilitates measurements for the 3D velocity distribution and mass spectrum for ions over a 1.5*π* field-of-view (FoV). It has an instantaneous FoV detection, which drastically improves the time resolution of the measurement, in comparison with the conventional FoV scanning method. PICAM’s covers ions up to ~3 keV energies, with mass range extending up to 132 a.m.u., offering a high mass resolution (e.g. differentiating between Mg^+^ and Na^+^ ions) needed for the MPO science objectives near Mercury (Orsini et al. 2021). Since PICAM is mounted on the side of MPO, during the cruise phase, it is predominantly pointing perpendicular to the Sun-line; meaning PICAM has restrictions for monitoring the Solar Wind. Nevertheless, PICAM has been switched ON for various cruise science, and outgassing investigation campaigns from 2021 onwards, and successfully collected observations (Alberti et al. 2023; Fränz et al. 2024; Riley et al. 2025). The best occasions for PICAM’s Solar Wind observations are when: (1) PICAM’s boresight makes the closest angle with MPO’s heliocentric velocity vector. This way the maximum number of ion fluxes are with PICAM’s field-of-view. (2) A structure in solar wind causes a thermally broader plasma to be observed (e.g. CMEs). In addition to that, whenever a SEP event arrives at MPO, depending on the energy of particles, PICAM may detect them as intensified background counts.

*SIXS*: Solar Intensity X-ray and particle Spectrometer (SIXS; Huovelin et al. 2020) onboard MPO measures high-energy electrons and protons with the SIXS-P particle detector, which consists of five orthogonal detectors made of 150 μm thick Si PIN diodes surrounding a CsI(Tl) scintillator with photodiode read-out. Each orthogonal detector defines one “Side” or field-of-view. It detects electrons in the range 50 keV to 3 MeV and protons in the range 1–30 MeV with a total nominal geometric factor of about 0.19 cm2 sr. It also measures X-rays with the SIXS-X detector, which consists of three almost identical silicon PIN diode detectors. The spectrum data are recorded in 265 energy channels in the energy range 1 keV–20 keV, and its last channel can be used as a particle detector. During the cruise, SIXS is in operation regularly except during solar electric propulsion arcs and power/technical constraints. Also, due to the cruise pack configuration of the mission, Sides 0 and 4 of SIXS-P are partially and totally obstructed by the spacecraft cruise shield.

*SPM*: The Solar Particle Monitor (SPM) onboard Mio is a particle detector that is part of the housekeeping sensors of MMO (Kinoshita et al. 2025). SPM performs the same function as BERM aboard the MPO spacecraft. It consists of two silicon photodiodes (SPM1 and SPM2) with an effective area of 10 mm × 10 mm and a depletion layer thickness of 0.3 mm. Each sensor has four different deposited energy channels. A magnesium case covers the silicon sensors, and the SPM is inside the Mio spacecraft. So SPM measures particles that have already decayed in energy and number after passing through the spacecraft’s structure. We derived each channel’s sensitive incident energy range and particle flux from such limited data acquired by SPM with Geant4 (Allison et al. 2016) radiation simulations. The calibrated data are consistent with BERM’s simultaneous data. Briefly, SPM sensors detect incident protons at 40–130 MeV, but we note that some contamination due to electrons is expected as described in Kinoshita et al. (2025). SPM is ready to be used as a particle observatory for BepiColombo, which has the highest measurement energy range. SPM operates only during specific solar wind or flyby campaigns during the cruise phase. SPM covers higher energy ranges than BERM and SIXS-P, so it plays an essential role in observing GCRs and associated phenomena such as Forbush decreases (Forbush 1938).

## Overview of coordinated observations during the cruise phase

BepiColombo has contributed to a variety of different topics during the cruise phase. Regarding the solar wind, although only the IMF is regularly measured, several studies have been performed. For example, Volwerk et al. (2023) investigated magnetic holes, which are widespread phenomena found in the solar wind and planetary magnetosheaths. They are characterised by a significant decrease of the magnetic field strength, balanced by an increased plasma pressure. Examining the occurrence of these magnetic holes between Earth and Mercury provides insight into the evolution of the solar wind. Using the magnetic field measurements of BepiColombo, Volwerk et al. (2023) found an almost constant occurrence rate for magnetic holes, indicating that the number of magnetic holes is constant through even larger heliospheric distances.

Regarding solar transient events, BepiColombo has also contributed to the global understanding of the internal structure of ICMEs (e.g. Kilpua et al., 2021), including their variability, propagation, evolution, erosion in time, and impact on planetary systems as it is following described. Möstl et al. (2022) followed several ICMEs from their launch time as detected by the Heliospheric Imager onboard STEREO-A to at least one of the following spacecraft: Solar Orbiter, BepiColombo, Parker Solar Probe, Wind, and STEREO-A. The paper includes 17 events in which four of them were observed by BepiColombo (20 April 2020, 29 May 2020, 29 June 2020, 8 October 2021). From these, there were also cases where the other spacecraft intercepted the same ICME. Weiss et al. (2021) also did a campaign between 19 April 2020 and 28 May 2020 in which they studied any CME directed to Earth by any other mission that could intercept them, including BepiColombo. They found a couple of CMEs in which they could reconstruct the flux rope of the CME at three different spacecraft locations simultaneously with their own flux rope model. Another study of particular interest in which a narrow separation of only 5° in longitude between BepiColombo, Solar Orbiter and STEREO-A encountered the same ICME was performed by Davies et al. (2020). The study showed clear flattening of the ICME cross-section and a dependence of the magnetic field strength that decreases slower with heliocentric distance than expected, as well as the expansion of the ICME being likely neither self-similar nor cylindrically symmetric. Another study was done by Palmerio et al. (2024a, b) who performed a similar analysis during the CME that occurred on 15 February 2022 and in which BepiColombo and Parker Solar Probe were at less than 0.03 AU of radial distance at the time of the CME-driven shock arrival in situ. This narrow separation, the shortest and closer to the Sun ever achieved in this kind of study, allowed the authors to investigate for the first time the mesoscale structure of a CME at Mercury’s orbit. The two sets of in situ measurements revealed some unexpected and profound differences, making the understanding of the overall 3D CME structure particularly challenging. This highlights the importance of solar wind monitoring at close distances from the Sun (i.e. Mercury’s distance), where the evolution of solar transients seems to be more dramatic.

Another important contribution of BepiColombo is the study of SEP events, which is especially important because the majority of these studies come from the radiation monitor, BERM, which was conceived as part of the housekeeping spacecraft suit but was upgraded to be part of the main scientific payload after launch. Since BERM is in operation continuously (see Fig. 2), with a few exceptions (see Pinto et al. 2022), it has significantly contributed to the understanding on how SEPs spread in the inner Solar System. For example, the first widespread event detected by BepiColombo occurred on 17 April 2021 (Dresing et al. 2023), being well observed by BepiColombo (BERM, SIXS and SPM), Parker Solar Probe, Solar Orbiter, STEREO-A, and near-Earth spacecraft, with BepiColombo being the best-connected mission. This extensive observation allowed for a very comprehensive timing analysis of the inferred solar injection times of the SEPs observed at each spacecraft, suggesting that different source processes were important for the electron and proton events. It also suggested a stronger shock contribution for the proton event and a more likely flare-related source for the electron event. Another example is the SEP event that occurred on 15 February 2022 (Khoo et al. 2024), where one of the most intense SEP events so far in Solar Cycle 25 was observed by Parker Solar Probe and BepiColombo (BERM), which also received the CME-driven shock, and by Solar Orbiter, STEREO-A, near-Earth spacecraft (e.g., Advance Composition Explorer (ACE), SOHO, and WIND), and Mars missions (e.g., the Mars Atmosphere and Volatile EvolutioN MAVEN, Mars Express and Mars Science Laboratory (MSL)), although affected by SIR-driven effects. This event allowed not only for characterising the propagation of the widespread SEP in the inner solar system, but to cross-calibrate BERM with similar instrumentation in Parker Solar Probe as both spacecraft were very close to each other.

Magnetic connectivity is an important topic, especially when it affects the propagation of SEPs. BepiColombo observations combined with modelling were key to characterise the effect of the ambient solar wind on a CME-driven shock and the associated gradual SEP event (Lario et al. 2022) that occurred on 9 October 2021. The presence of a high-speed solar wind stream affected the propagation of low energy ions (5 MeV) of the gradual SEP event, connecting two regions, BepiColombo and Earth when a priori they should not be magnetically connected. Wijsen et al. (2023) performed a simulation with the magnetohydrodynamic model EUHFORIA (European Heliospheric FORecasting Information Asset, Pomoell and Poedts 2018) and the energetic particle transport model PARADISE (PArticle Radiation Asset Directed at Interplanetary Space Exploration), demonstrating the influence of even modest background solar wind structures on the development of SEP events.

Furthermore, BepiColombo has been used as an upstream solar wind monitor for other planets, such as Mars, which despite its current robotic exploration, do not have continuous upstream solar wind observations, making Mars space environment analyses very challenging (e.g. Sánchez-Cano et al. 2022). In this respect, Chi et al. (2023) analysed the dynamic evolution of a couple of ICMEs in 2021 from the Sun up to Mars by combining observations from BepiColombo upstream Mars, and Tianwen-1 and MAVEN at Mars. Having these upstream observations was key to understanding the response of the Martian ionosphere to ICMEs, which are one of the causes for Mars Space Weather (Yu et al. 2023). Furthermore, BepiColombo has significantly contributed to disentangling one of the major Space Weather issues at Mars. A high energetic proton-rich SEP event hit Mars creating one of the largest ever recorded ground level enhancements (Khoo et al. 2024). This event, however, does not create major high-frequency disturbances in the ionosphere, which is the opposite behaviour of what typically happens during major events of this type (e.g., Sánchez-Cano et al. 2019; Lester et al. 2022). Thanks to BepiColombo being upstream of Mars, we have been able to disentangle the effect of the SEP particles in the ionosphere and contribute towards the understanding of the reaction of the Martian system to Space Weather activity (Khoo et al. 2024). Beyond Mars, BepiColombo was included in a multi-mission study to characterise how Jovian electrons propagate in the inner heliosphere. Strauss et al. (2024) did a long-term analysis of high-energy particles detected by Parker Solar Probe, Solar Orbiter, STEREO-A, BepiColombo, SOHO and MAVEN, and reported that it was confirmed that Jovian electrons could reach small heliocentric distances without being completely impeded by the outward moving solar wind, but they are difficult to observe.

Regarding the combination of in situ and remote observations, radio emissions from extragalactic radio sources can be scattered by the disturbances included in the solar wind, which is observed as the interplanetary scintillation (IPS). The IPS has been an important tool to reconstruct the global structure of the solar wind (Kojima et al 1998). The IPS data are also used to detect the CMEs propagating in the interplanetary space (Iwai et al 2019), especially the sheath of CMEs, as they are dense regions that can be formed in front of a fast-propagating CME. This sheath region can significantly scatter radio emissions. The cross-correlation of the IPS signal observed in spatially separated stations at Earth can also give the propagation speed of the solar wind (Kojima et al 1998). The derived solar wind density and speed data can be projected onto the solar source surface using the tomography technique (Jackson et al 1998) that enables us to understand the global structure of the heliosphere. Jackson et al (2023) investigated a CME observed on 10 March 2022. They reconstructed the 3D structure of the CME by the IPS observation data of ISEE, Nagoya University and its tomography analysis, and explained the CME structures observed in situ by a number of spacecraft including BepiColombo.

Solar wind observations are typically combined with magnetohydrodynamic (MHD) solar wind simulations, such as Enlil (Odstrcil 2003), SUSANOO (Shiota and Kataoka 2016), and EUHFORIA. All of them have enabled us to estimate CME propagation and their interactions with background solar wind. For example, SUSANOO is a MHD simulation of the inner heliosphere to simulate the propagation of solar wind and multiple CMEs which have twisted magnetic structure inside (Shiota et al 2014; Shiota and Kataoka 2016). The global MHD simulation can be improved by including the IPS data. For example, Jackson et al (2015) used the IPS data as the inner boundary of Enlil, while Iwai et al (2021) used the IPS data to optimise the location of the CME front in the SUSANOO-CME simulation. The global MHD simulation together with the coronagraph and IPS data can provide the simulated in situ data at the BepiColombo location. In another study, Volwerk et al (2021) used the SUSANOO simulation to estimate the solar wind during the first Venus flyby, which was essential to understand the flapping of the Venusian tail with distance.

BepiColombo has already contributed to a significant number of studies as described in this section, but more science is expected to come as the majority of the dataset is yet unexploited as it will be shown in the next two sections.

## Multi-instrument observations of transient solar events from end-2018 until mid-2024

**Fig. 3:**
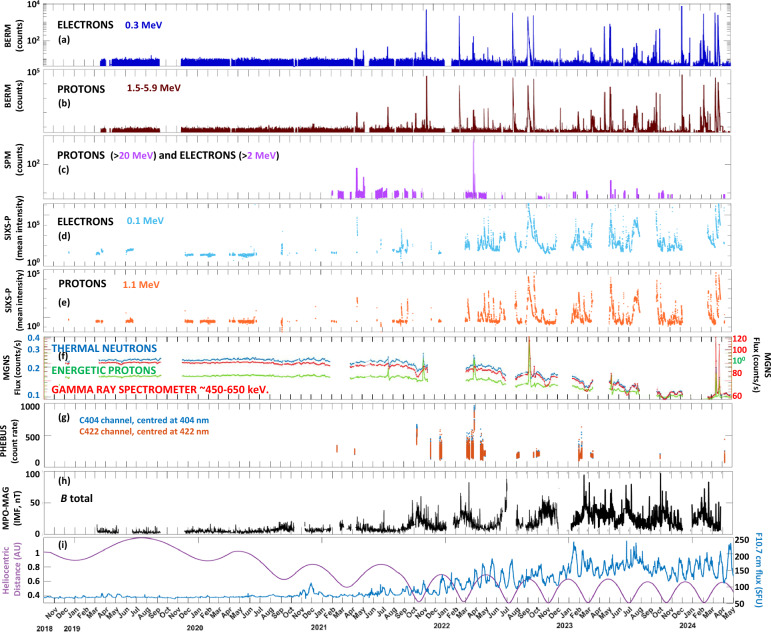
BepiColombo observations of energetic particles and interplanetary magnetic field since launch until May 2024. **a**,**b** Energetic electrons and protons measured by BERM in the indicated energy channels, respectively. **c** Energetic electrons and protons (mixed) measured by SPM in the indicated energy channel. **d**,**e** Energetic electrons and protons, respectively, measured by SIXS-P in the indicated energy channels. **f** MGNS thermal neutrons, energetic protons and gamma ray spectrometer data in the indicated energy range. **g** PHEBUS observations of the dark current in visible channels (proxy for SEP detections). **h** Interplanetary magnetic field (IMF) intensity measured by MPO-MAG. **i** Heliocentric distance (purple) and solar flux (F10.7 cm, blue) covered by BepiColombo during the cruise phase until May 2024

The overview of BepiColombo cruise observations from the particle and magnetic field instruments is shown in Fig. 3. In particular, we show electron and proton data (if available) for the lower energy channels of BERM, SPM, and SIXS-P (panels a–e, respectively). In panel f, we show data from three channels from the MGNS instrument. In particular, we show energetic protons, thermal neutrons and data from the gamma ray spectrometer. The three channels are sensitive to SEP arrivals and can also record them, although we note that MGNS does not necessarily detect the same number of events than BERM, SPM or SIXS-P as MGNS SEP detections come from their background counts (see Sect. 2). We also show observations of the dark current of the visible channels of the PHEBUS instrument, which has been used during the cruise for calibration purposes during some specific campaigns and from which we can infer SEPs detections. These are the visible channels c404 that is centred at 404 nm and the c422 channel that is centred at 422 nm. These observations were executed at the parking position of the instrument, implying that no light was entering the baffle. As can be seen in panel g, there are two series of observations, one in October 2021 and the other one in March 2022, which agree very well with the particle instruments, indicating that they are the product of two large SEP events that hit the spacecraft, which in turn give us an idea of the minimum energy for the instrument to detect SEP particles in the detectors. We also show the total magnetic field as recorded by MPO-MAG in panel h, and the heliocentric distance and solar flux covered by BepiColombo during the cruise (extracted from Fig. 1) in panel i. Figure 3 covers 5.5 years of observations, from launch until beginning of May 2024 when an anomaly in the transfer module was detected.^1^ We decided to stop this Figure and also the events recorded in Table 1 in May 2024 as after this period, observations are highly constrained by the power onboard the spacecraft. However, we note that some data are available after operations were resumed in August 2024 as seen in Fig. 2, although not at the same cadence as before (controlled by power limitations).

**Table 1:** SEP events detected by BepiColombo

SEP event (#)	Start day	Type of event	Instruments that detected the event	Notes
1	08 Dec 2020	Proton event	BERM	Minor event Event studied by Palmerio et al. (2022)
2	17 Apr 2021	Proton and electron event	BERM, SIXS, SPM, MGNS	First clear major event detected by BepiColombo Event studied by Dresing et al. (2023)
3	07 May 2021	Proton and electron event	BERM, SPM, MGNS	
4	17 May 2021	Proton event	MGNS	
5	13 Jun 2021	Proton event	BERM	
6	16 Jul 2021	Electron event	BERM	
7	18 Jul 2021	Proton and electron event	BERM	
8	26 Aug 2021	Proton event	SIXS	
9	13 Sep 2021	Proton event	BERM, SIXS, MEA	
10	17 Sep 2021	Proton event	MGNS	
11	28 Sep 2021	Proton event	BERM, MGNS	
12	01 Oct 2021	Proton event	MGNS	
13	09 Oct 2021	Proton and electron event	BERM, PHEBUS, MGNS	See Sect. 5.2 Event studied by Palmerio et al. (2024b), Wijsen et al. (2023), Lario et al. (2022)
14	28 Oct 2021	Proton and electron event	BERM, MGNS	
15	30 Oct 2021	Proton and electron event	BERM, MGNS	
16	02 Nov 2021	Proton and electron event	BERM	
17	09 Nov 2021	Proton and electron event	BERM	
18	10 Nov 2021	Proton event	MGNS	
19	20 Nov 2021	Proton event	BERM	
20	15 Feb 2022	Proton and electron event	BERM, housekeeping	See Sect. 5.5 Event studied by Khoo et al. (2024), Palmerio et al. (2024a), Sánchez-Cano et al. (2023)
21	10 Mar 2022 12 Mar 2022	Proton event	BERM, SIXS, MSA, MIA, MEA, MGNS, MIPA*	SPM detected a FD (12 Mar 2022) See Sect. 5.3 Event studied by Jackson et al. 2023 *MIPA had an increase on 11 Mar 2022 PICAM has data on the 11th and 12th
22	28 Mar 2022	Proton and electron event	BERM, SPM, PHEBUS, MEA, MSA, MGNS, PICAM, MIPA	SPM detected a FD in addition to the SEP See Sect. 5.4
23	30 Mar 2022	Proton and electron event	BERM, SPM, MEA, MSA, MGNS	See Sect. 5.4
24	31 Mar 2022	Electron event	BERM, MEA, MSA, HEP-e, MGNS	See Sect. 5.4
25	02 Apr 2022	Proton and electron event	BERM, SPM, MEA, HEP-e, MGNS	See Sect. 5.4
26	20 Apr 2022	Proton event	MGNS	
27	29 Apr 2022	Proton and electron event	BERM, SIXS, MGNS	PICAM has data
28	11 May 2022	Proton and electron event	BERM, SIXS, MGNS	
29	25 May 2022	Proton and electron event	BERM, SIXS	
30	14 Jun 2022	Proton event	BERM, SIXS	
31	26 Jun 2022	Proton event	SIXS	
32	23 Jul 2022	Proton and electron event	BERM	
33	31 Jul 2022	Proton and electron event	BERM, SIXS, MGNS	
34	04 Sep 2022 06 Sep 2022	Proton and electron event	BERM, SIXS, MGNS	PICAM has data Event studied by Riley et al. (2025)
35	23 Sep 2022	Proton and electron event	BERM, SIXS, MGNS	
36	29 Sep 2022	Electron event	BERM, SIXS, MGNS	
37	11 Dec 2022	Proton and electron event	BERM	Start of the event not visible
38	12 Dec 2022	Proton event	BERM	
39	17 Dec 2022	Proton and electron event	BERM	
40	14 Jan 2023	Proton event	SIXS, MGNS	
41	18 Jan 2023	Proton event	SIXS	
42	02 Feb 2023	Proton and electron event	BERM, SIXS, MGNS	PICAM has data
43	17 Feb 2023	Proton and electron event	BERM, SIXS, MGNS	
44	24 Feb 2023	Proton event	MGNS	
45	06 Mar 2023	Proton event	SIXS	
46	12 Mar 2023 13 Mar 2023 14 Mar 2023	Proton and electron event	BERM, SIXS, MGNS	
47	22 Mar 2023	Proton and electron event	BERM	
48	09 Apr 2023	Proton and electron event	BERM	
49	21 Apr 2023	Proton and electron event	BERM	
50	07 May 2023 09 May 2023 10 May 2023	Proton and electron event	BERM, SIXS, MGNS	
51	16 May 2023	Proton and electron event	BERM, SIXS, MGNS	
52	01 Jun 2023	Proton event	BERM, SIXS	
53	13 Jun 2023	Electron event	SIXS	
54	14 Jun 2023	Proton and electron event	SIXS	
55	16 Jun 2023	Proton and electron event	BERM, SIXS, MGNS	
56	19 Jun 2023	Proton event	MGNS	
57	22 Jun 2023	Proton event	BERM, MGNS	Start of the event not visible
58	10 Jul 2023 13 Jul 2023 14 Jul 2023	Electron and proton event	SIXS, MGNS	Electron event starts on 10 July and Proton event starts on 14 July. Several SEP electron injections on 14 July
59	17 Jul 2023 22 Jul 2023 23 Jul 2023 26 Jul 2023 28 Jul 2023	Proton and electron event	BERM, SIXS, MGNS	
60	12 Aug 2023	Proton and electron event	BERM	
61	27 Aug 2023	Proton and electron event	BERM	
62	31 Aug 2023	Proton and electron event	BERM	
63	06 Sep 2023	Proton event	BERM	
64	08 Sep (protons and electrons) 2023 13 Sep (electrons) 2023 16 Sep (protons) 2023	Proton and electron event	BERM	
65	21 Sep (protons and electrons) 2023 22 Sep (protons) 2023 24 (electrons) 2023	Proton and electron event	BERM, SIXS, MGNS	
66	01 Oct 2023 02 Oct 2023	Electron event	SIXS	
67	03 Oct 2023 04 Oct 2023	Proton and electron event	BERM, SIXS, MGNS	
68	09 Oct 2023 10 Oct 2023	Electron event	SIXS	PICAM has data, but only solar wind observed
69	16 Nov 2023	Proton and electron event	BERM, SIXS	
70	04 Dec 2023	Proton event	MGNS	
71	07 Dec 2023 09 Dec 2023	Proton and electron event	BERM	PICAM has data
72	12 Dec 2023	Proton event	BERM	PICAM has data, but only solar wind observed
73	29 Dec 2023	Proton and electron event	BERM	
74	31 Dec 2023	Proton and electron event	BERM	
75	30 Jan 2024	Proton and electron event	BERM	
76	02 Feb 2024	Proton and electron event	BERM	
77	09 Feb 2024 10 Feb 2024 13 Feb 2024	Proton and electron event	BERM	
78	21 Feb 2024	Proton and electron event	BERM, SIXS, MGNS	
79	28 Feb 2024	Proton and electron event	BERM, SIXS	
80	01 Mar 2024	Electron event	SIXS	
81	02 Mar 2024	Electron event	SIXS	
82	07 Mar 2024 08 Mar 2024	Electron event	SIXS, MGNS	
83	10 Mar 2024	Proton event	MGNS	
84	15 Mar 2024	Proton and electron event	BERM, SIXS, MGNS	
85	23 Mar 2024	Proton and electron event	BERM, SIXS, MGNS	
86	20 May 2024	Unknown	Housekeeping	See Sect. 5.5

As can be seen in Fig. 3, the solar cycle clearly controls all observations taken during the cruise. Starting with the particles, each spike seen in panels a-e corresponds with a SEP event detected by the mission. The first large SEP was detected in April 2021, which was also a very well spread SEP event detected by most of the missions currently in space (Dresing et al. 2023). Since then, the number of SEP detections has progressively increased both in frequency and in intensity, in accordance to the rise of the solar activity seen in panel g. The start dates of these events, as well as which instrument detected it, are shown in Table 1. We only indicate the day of the arrival time as each instrument is sensitive to different energies and therefore, arrival times may slightly differ from one to another dataset, as well as their intensities. Table 1 and Fig. 3 show that BepiColombo have observed 86 SEP events from launch until May 2024, 50 of them showing significant enhancements in the proton and electron fluxes, 26 of them showing only proton enhancements, 9 of them showing only electron enhancements, and 1 of them is not known as it was detected only with housekeeping data from the spacecraft (see Sect. 5.5). We note that those SEP events that were only detected by protons or electron channels in the instrumentation are typically quite minor-moderate events.

Moreover, if we focus on the background noise, mainly from BERM (panels a-b) and MGNS (panel f) observations, one can see that the noise level has a subtle variability, being more particles observed during the first part of the cruise and less during the second part, reaching a minimum towards the end of the figure. This is simply the effect of solar-cycle dependent GCR modulation (Pinto et al. 2025). Although BERM has no energy resolution to detect direct GCR fluxes, it can measure the secondary particles produced on the spacecraft by GCR impacts. This anticorrelation in the GCR-proxy detections and the solar cycle is a well-known phenomenon. Such anticorrelation has also been observed in sporadic observations by BELA and MIXS, which are presumably sensitive to GCRs. However, there are not many observations of GCRs at closer distances from the Sun. BepiColombo’s trajectory in the inner heliosphere is unique in the sense that it allows us to investigate the effect of both the solar cycle and the heliocentric distance on GCR propagation. A more detailed study in this topic is currently being performed, and it will complement similar observations at further distances performed by missions such as Rosetta in the outer heliosphere (Honig et al. 2019).

The solar cycle is also clearly seen in the IMF observations recorded by MPO-MAG during the cruise. The variability, as seen as small spikes in the data, corresponds to solar transients, such as CMEs, seen during larger levels of solar activity. However, the IMF is not only influenced by the solar activity. The distance of BepiColombo with the Sun plays a major role in the modulation of this parameter. If panels h and i (purple line) are compared, one can see a clear anticorrelation of the IMF intensity with the distance to the Sun. The IMF intensity decreases with the square of the distance from the Sun, being on average ~7 nT at Earth distances, and ~45 nT at Mercury distances. When the mission encounters transients like CMEs at closer distances to the Sun, the recorded IMF can easily rise to ~100 nT.

## Highlights of specific solar events

Due to the nature of the cruise, not all the instruments can be in operation simultaneously. In this section, we provide a few science highlights based on particular solar events that were detected by different BepiColombo instruments and techniques.

### Remote observations of the solar wind near Sun’s surface (11–13 solar radii) and solar corona on 13–14 March 2021

While not the primary objective of the MORE experiment, this instrument can investigate the plasma properties of the solar wind in the inner heliosphere, including the solar corona, when BepiColombo is in a superior solar conjunction (i.e. BepiColombo is occulted by the Sun as seen from Earth). This is an interesting way of getting observations very close to the Sun’s surface, which is a region from which few observations are available. During this type of measurements, the radio link sent by BepiColombo towards Earth propagates through the solar corona and solar wind, and these mediums produce perturbations in the radio signals, from which the velocity, density, and turbulent spectrum of the solar wind properties crossed by the radio signal can be retrieved. Furthermore, Doppler data collected during BepiColombo solar conjunction can be analysed to pinpoint plasma structures along the line of sight, such as coronal mass ejections.

**Fig. 4:**
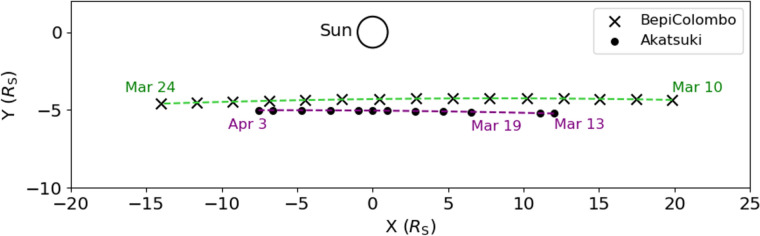
Relative positions of BepiColombo (crosses) and Akatsuki (dots) on March–April 2021 as seen from Earth.

In March 2021, BepiColombo and JAXA’s Venus orbiter Akatsuki (Imamura et al. 2011; 2017) were in the superior solar conjunctions, and, notably, the positions of these spacecraft, as seen from the Earth, were almost overlapped. Furthermore, on 13 and 14 March, the observational points of both BepiColombo and Akatsuki were aligned almost radially with respect to the Sun. Figure 4 shows the positions of BepiColombo and Akatsuki as seen from Earth. The observations by BepiColombo covered heliocentric distances of 8–12 solar radii, and the ray path passed the southern side of the Sun. Most of BepiColombo and Akatsuki’s observations during the 2021 campaign were conducted independently except for passes on 13 and 14 March, which corresponded to a simultaneous observation.

BepiColombo conducted a simultaneous radio experiment with Akatsuki to trace the radial development of the solar wind. When the radio signals of both missions cross the same solar wind stream, significant patterns appear on both BepiColombo and Akatsuki Doppler-shift time series. These patterns allow us to calculate a time lag between two signals, representing the travel time of passively advected plasma. Then, we can obtain the solar wind velocity by dividing the radial distance between the tangential points of the two spacecraft’s ray paths (Cappuccio et al. 2024). Table 2 summarises the information about the data set exploited and the geometry of the experiments (Cappuccio et al. 2024). It is important to note that the radio tracking passes of both spacecraft overlapped for about 3.6 h on 13 March and 1.7 h on 14 March. Table 2 also shows the distance between the observational points, 1.7 solar radii on 13 March and 3.2 solar radii on 14 March. Important to note that while Akatsuki used an X-band (~8.4 GHz) downlink signal transmitted by the onboard Ultra Stable oscillator (USO), the MORE experiment used a multi-frequency radio link configuration (see Sect. 2).

**Table 2:** Summary of the simultaneous observations during 13 and 14 March 2021 by BepiColombo/MORE and Akatsuki missions (table from Cappuccio et al. 2024)

	13 March 2021 (UT)	14 March 2021 (UT)
BepiColombo pass	13:35–19:10	13:35–19:10
Akatsuki pass	13:35–19:10	13:35–19:10
Overlap	3.6 h	1.7 h
Spacecraft relative radial distance	1.7 *R*_S_	3.2 *R*_S_
Angular separation	1.7°	2.7°
Solar latitude	−22°	−26°
Spacecraft distance from the Sun	0.52 AU	0.73 AU

**Fig. 5:**
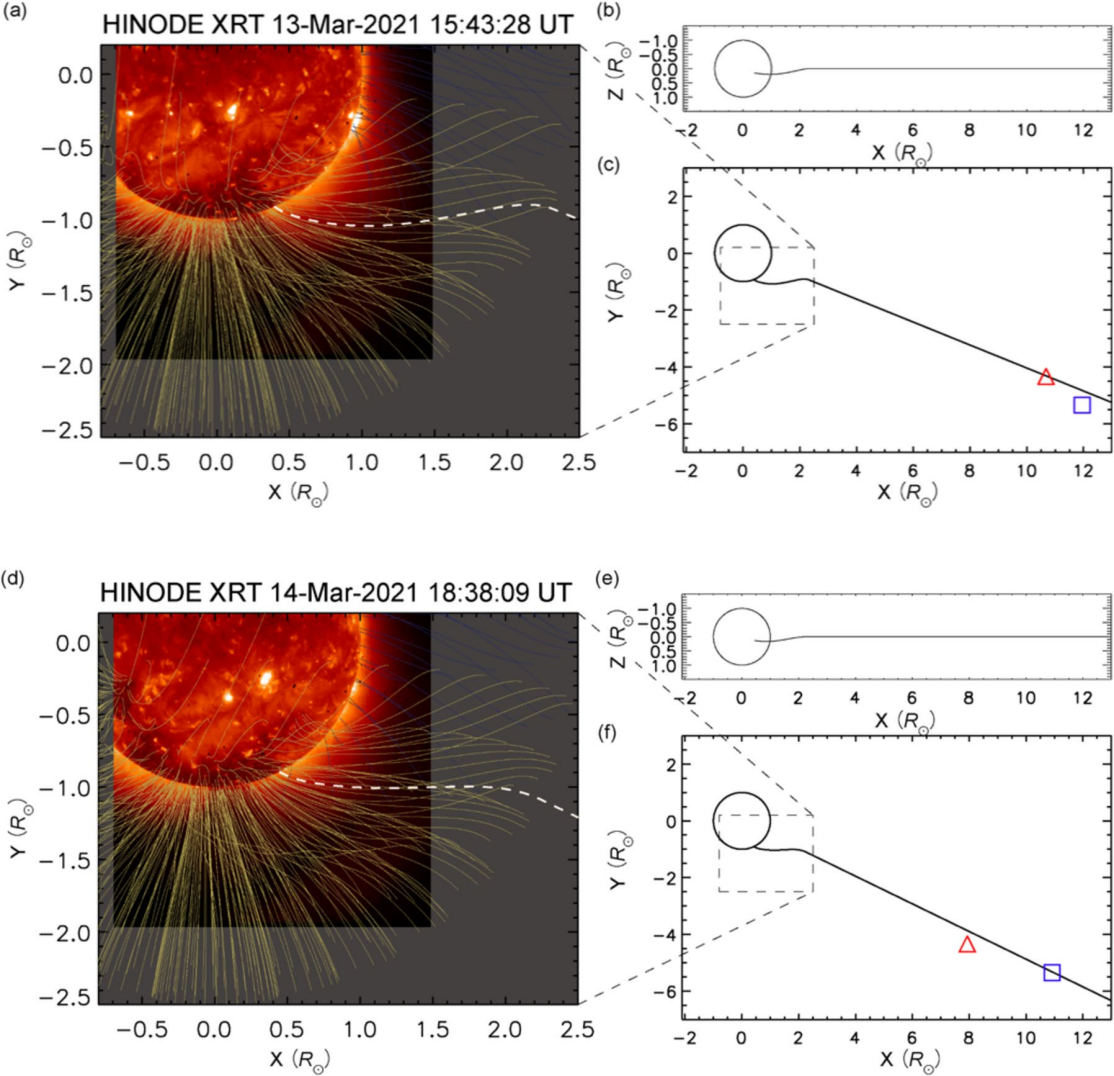
Connection between magnetic fields and the ray path on 13 and 14 March 2021 (figure from Cappuccio et al. 2024). Panels **a** and **d** show potential magnetic field lines on March 13 and March 14 extrapolated from a magnetic field map observed by HMI/SDO (Scherrer et al. 2012; Schou et al. 2012), superposed on an X-ray image taken by Hinode XRT (Golub et al. 2007). The white dashed line on panels **a**/**d** shows the field line that crosses the observational points, and black lines in panels **c**/**f** correspond to the same magnetic field lines. Here, the red triangle indicates BepiColombo and the blue square indicates Akatsuki spacecraft, respectively. Panels **b**/**e** show the same magnetic fields as panels **c**/**f** seen from the different plane. The *X*-axis is directed to the western limb, the *Y*-axis is directed to the northern pole, and the *Z*-axis is directed to Earth.

During this experiment, Cappuccio et al. (2024) found that the two Doppler data on 13 March clearly correlate at a time-lag of 2910 s. Using the knowledge of the relative distance between the two probe-ground station lines of sight at the closest approach to the Sun, it was estimated that the solar wind velocity was 421 ± 21 km/s (at 10–12 solar radii). Following the same procedure for the second experiment on 14 March, it was estimated that the solar wind speed velocity was 336 ± 7 km/s (at 8–11 solar radii). These velocities correspond to a typical velocity of well-developed slow winds. Furthermore, the analysis of the magnetic fields and the X-ray image show that the field lines connected to the observational points originated from the vicinity of the outer edge of the prominent polar coronal hole (Fig. 5) (Cappuccio et al. 2024). It is known that the boundary of the coronal hole has been suggested as a possible candidate of a source of the slow solar wind (e.g., Wang and Sheeley 1990; Wang and Ko 2019; Viall and Borovsky 2020). Therefore, according to the configuration of the magnetic fields, the observations on March 13 and 14 may have probed the slow winds from the boundary regions.

A different study can be performed by analysing MORE Doppler data only, to obtain the space–time localisation of plasma structures along the line of sight, such as CMEs. Microwave signals are continuously exchanged between the ground station and the spacecraft. A plasma concentration located at a specific distance $x_{{\text{P}}}$ along the line of sight from the Earth station, induces phase perturbations on both the uplink and downlink signals, causing two distinct signatures in the two-way tracking data (RiChie-Halford et al. 2009). The temporal gap between these plasma-related occurrences in the Doppler time series is $\tau_{{\text{s }}} = t_{{{\text{RTLT}}}} - 2x_{{\text{P}}} /c ,$ where $t_{{{\text{RTLT}}}}$ is the round-trip light time. $\tau_{{\text{s }}}$ can be measured by computing the cross-correlation between uplink and downlink plasma-induced fluctuations, identifying the time lag corresponding to a peak in the cross-correlation function. This method enables the determination of $x_{{\text{P}}}$, the plasma feature’s distance from Earth, facilitating the temporal and three-dimensional spatial localisation of plasma events. In the simplified case of a geometrically thin plasma screen, the uplink and downlink signals intersect at the same point along their paths, leading to identical phase shifts in both signals. As a result, the Doppler time series for uplink and downlink are exact time-shifted replicas, producing a cross-correlation function that peaks at a value of one at a time lag of $t_{{{\text{RTLT}}}} - 2x_{{\text{P}}} /c$. However, if the plasma screen has measurable thickness, the segments of the plasma traversed by the uplink and downlink signals differ. Consequently, their Doppler time series no longer perfectly mirror each other, and the peak value of the cross-correlation function drops below one. The reduction from unity in the cross-correlation function indicates the effect of the plasma screen’s thickness on the signals.

**Fig. 6:**
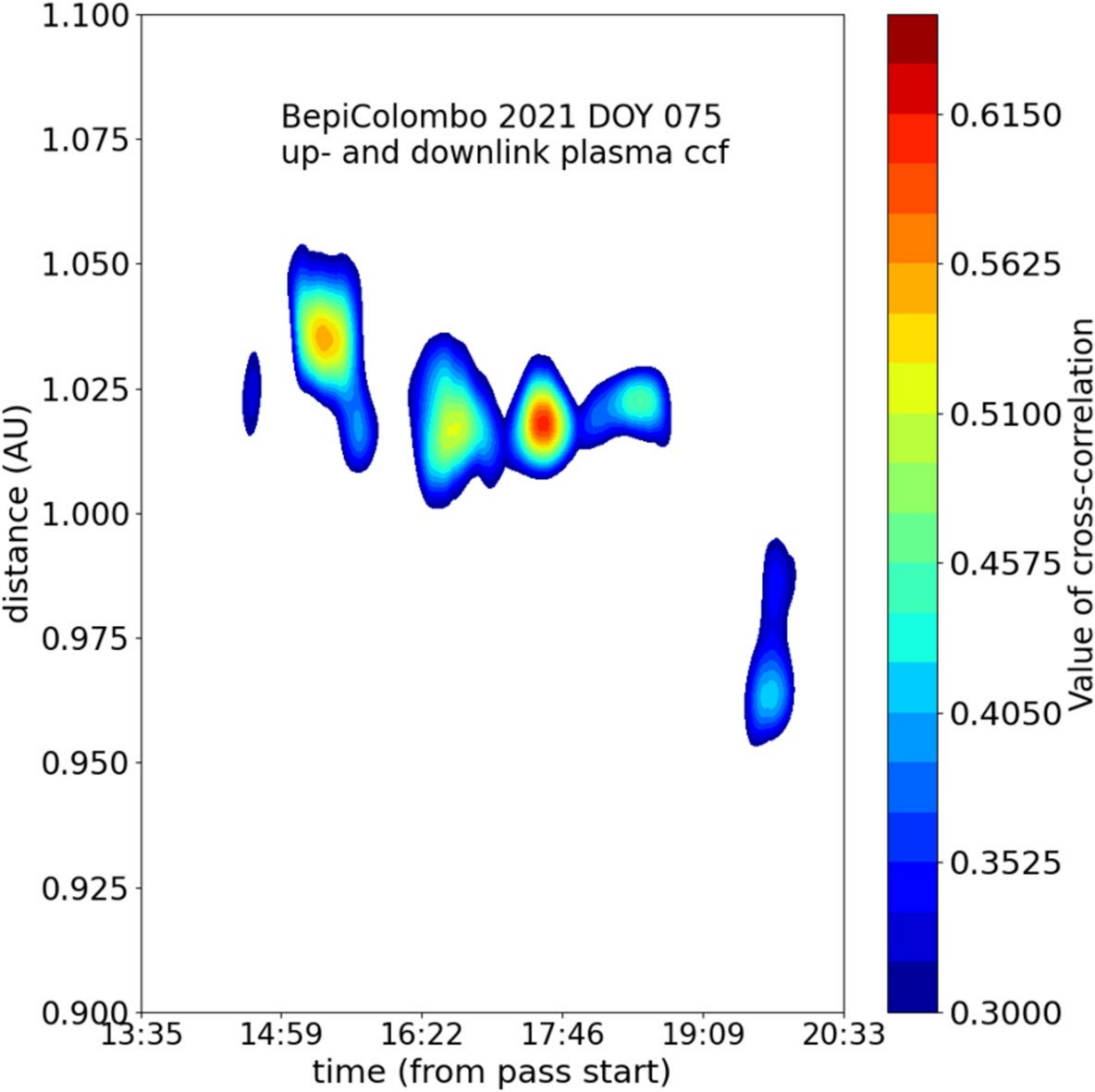
Cross-correlation for locating the CME on 16 March 2021 (di Stefano et al. 2024). The horizontal axis represents the time (UT) during the observation, while the vertical axis indicates the distance from the Earth. Here, 1.0 AU roughly corresponds to the position of the Sun. The contour shows the value of the cross-correlation coefficient calculated from the uplink and downlink signals.

MORE X/X, X/Ka and Ka/Ka Doppler data can be combined to provide, separately, the non-dispersive measurement and the plasma-induced fluctuations on the uplink and downlink signals. Analysing these plasma-induced fluctuations with this technique, it was possible to pinpoint a CME that was detected during MORE observations on 16 March 2021, when the spacecraft was in a superior solar conjunction. The white-light images observed by the SOHO/LASCO coronagraph indicated that this CME crossed the line-of-sight of the ray path of the radio signal on 16 March 2021. The presence of this CME along the path of the signal was confirmed by the detection of a peak in the cross-correlation between the uplink and downlink plasma-induced fluctuations in MORE Doppler data. The results of this analysis for the radio tracking pass performed on 16 March 2021 are reported in Fig. 6, showing the space–time cross-correlation function of plasma uplink and downlink time series; the y-axis is the distance from the Earth; the x-axis is downlink received time. The peak of the cross-correlation value indicates that the CME crossed the path of the radio signal four hours after the beginning of the pass, at 1.02 AU from Earth.

### CME structure analysis on 9 October 2021 detected with in situ instruments

**Fig. 7:**
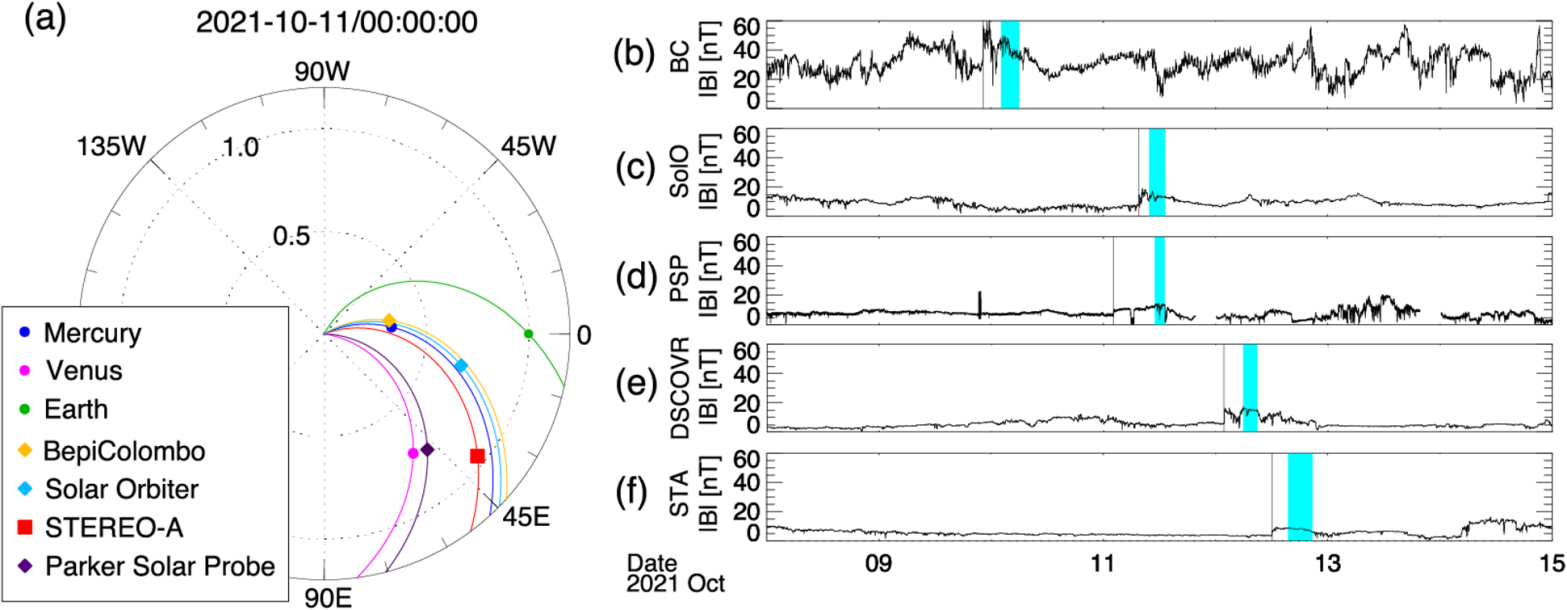
**a** The planetary and spacecraft location at 00:00 UT on 11 October 2021. Each coloured symbol denotes the individual spacecraft and planet locations summarised in the bottom left legend. **b**–**f** The time profile of magnetic field strength obtained from individual spacecraft. The vertical solid lines in each panel indicate the shock arrival. The time interval that we mainly analysed in this study is highlighted in cyan.

BepiColombo observed a CME event from 9 to 10 October 2021. This CME was associated with an M1.6-class solar flare that occurred at 06:19 UT on 9 October in the solar active region NOAA 1882, which was located in the northern hemisphere and near the central meridian of the Sun. Figure 7a shows the overview of the planetary and spacecraft alignments viewed from the inner heliosphere during the CME passage. BepiColombo observed the CME at 0.33 AU from the Sun and N2.2 latitude and W2.0 longitude. Multiple spacecraft, including Solar Orbiter, Parker Solar Probe, STEREO-A, and Deep Space Climate Observatory (DSCOVR), also observed the CME signature at different locations in the inner heliosphere, although all the spacecraft observed a flank of the CME because the CME erupted toward the northwest from the Sun. By analysing MPO-MAG data, an interesting flip signature in the magnetic field was found, which is also seen in at least four spacecraft. A future study will further investigate the ICME event in October 2021 to understand these signatures by analysing in situ data from the four missions described above.

**Fig. 8:**
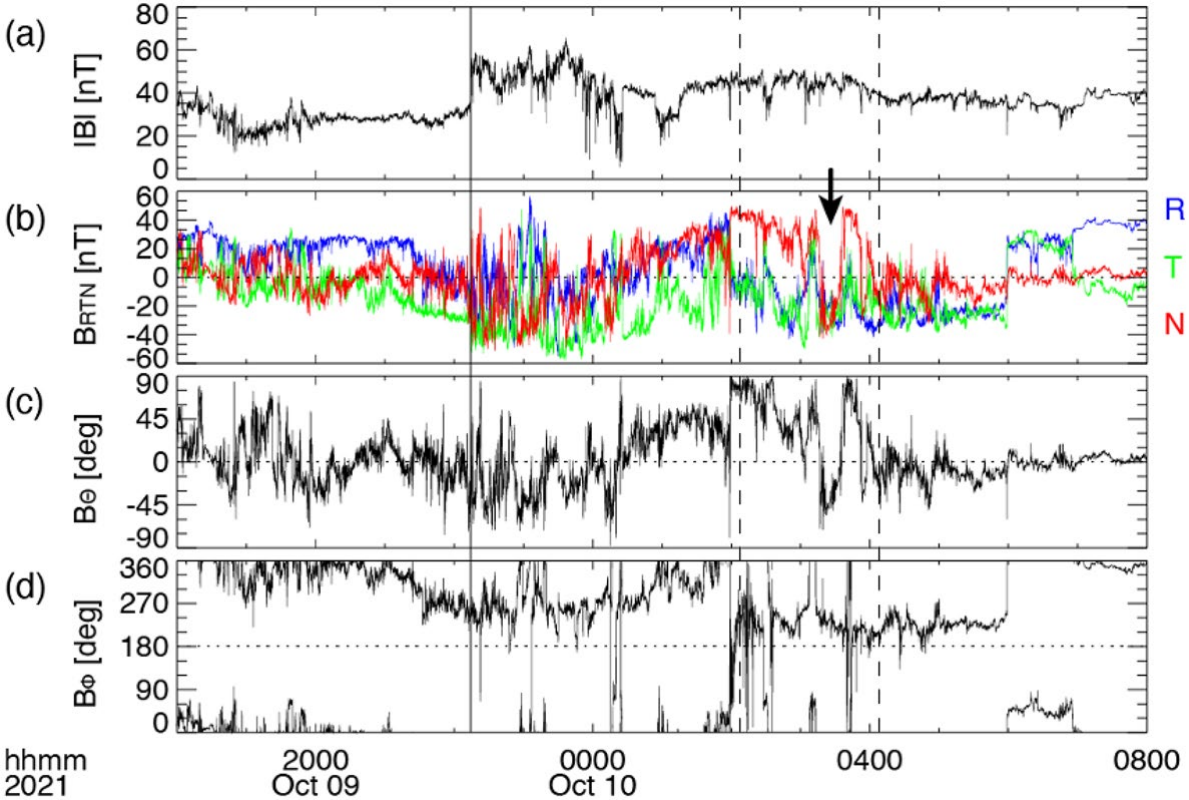
Summary of the in situ magnetic field measured by BepiColombo/MPO-MAG from 18 UT on 9 October to 08 UT on 10 October 2021. The vertical solid line indicates the shock arrival time at 22:14:06 UT on 9 October. The vertical dashed lines are the time range of 02:07:30–04:08:10 UT on 10 October, where we focus the study. The time-series plots of the **a** magnetic field magnitude, **b** vector magnetic field in the spacecraft-centred radial–tangential–normal (RTN) coordinates; red, green, and blue correspond to the radial, tangential, and normal components, and the magnetic field **c** elevation and **d** azimuth angles in the spacecraft-centred RTN coordinates. The black arrow in panel **b** depicts the flip in the magnetic field normal component (panel **b**, see text for explanations), which we focused on in the present study

The magnetic field observed by BepiColombo/MPO-MAG is shown in Fig. 7b. The vertical black solid line represents the interplanetary shock arrival at BepiColombo at 22:14 UT on 9 October. When BepiColombo was in the sheath downstream of interplanetary shock, we found the outstanding flip in the magnetic field normal component, which is indicated by the black arrow in Fig. 8b and has the following characteristics: the magnetic field strength of the normal component *B*_n_ temporarily drops and flips its sign. The flip signature is observed for several tens of minutes. Such a flip in the magnetic field is also seen in the magnetic field profiles obtained from Solar Orbiter, STEREO-A, and DSCOVR hatched by cyan in Fig. 7b–f, although it is vague in the Parker Solar Probe profile. A minimum variance analysis (Sonnerup and Scheible 1998) has been applied to the in situ magnetic field data obtained from the multiple spacecraft to understand the structure leading to the flip signature. We selected the time interval including the flip signature indicated by the vertical dashed lines in Fig. 8 and shaded by cyan in Fig. 7b–f and where the eigenvalue ratio between the intermediate and the minimum variance directions is larger than 2 following Ruan et al. (2023). Generally speaking, if the structure is due to the magnetic flux rope, the hodogram should be a simple circular shape in the plane perpendicular to the minimum variance direction (*B*_k_), in other words, a unimodal and bipolar peak signature should be seen in the maximum (*B*_i_) or intermediate (*B*_j_) variance directions depending on the crossing geometry. On the other hand, if the structure is due to the planar magnetic structure (PMS) in the CME-driven sheath (Nakagawa et al. 1989), the vector magnetic field should be just distributed on the plane perpendicular to the minimum variance direction (*B*_k_). Our analysis shows that the vector magnetic field is well distributed on the plane perpendicular to *B*_k_, which is consistent with the characteristic of PMS (not shown here).

We further estimated the shock normal direction *n* at each spacecraft location based on the coplanarity theorem (e.g., Kataoka et al. 2005) in order to discuss the spatial distribution of the flip in the magnetic field, i.e. that of the PMS in the CME-driven sheath in the inner heliosphere. The result presents that the angle between *B*_k_ and *n* is approximately 20° at Solar Orbiter, suggesting that the PMS is distributed along the shock surface. The angle between *n* and *B*_k_ in Parker Solar Probe and STEREO-A are nearly 45°, presumably owing to Parker Solar Probe and STEREO-A slightly longitudinally off from the Sun–Earth line. Although BepiColombo and DSCOVR are radially aligned to the Sun–Earth line as well as Solar Orbiter, the angles at DSCOVR and BepiColombo are estimated to be ~41° and rather large (~77°), respectively. In order to consider the discrepancy, we should note here that (1) there is some ambiguity in estimating the shock normal direction because the magnetic field is fluctuating in the downstream of the interplanetary shock, especially seen in BepiColombo (see Fig. 8); (2) the central part of the CME did not pass through the spacecraft in this event.

Unfortunately, no plasma observation by BepiColombo was available for the time interval of our interest, but we estimated the Alfvén Mach number *M*_A_ at each spacecraft located in the different inner heliosphere. Oliveira (2023) reported *M*_A_ observed at the interplanetary shock at 1 AU typically ranging between 1.74 and 4.05. However, the estimated *M*_A_ is as high as approximately 9 at DSCOVR and those are even larger at the smaller radial distance in the inner heliosphere. According to Hanneson et al. (2020), the magnitude of the magnetic field |*B*| is proportional to *r*^−1.5^, where *r* is the heliospheric radial distance, while the solar wind number density *N* is generally proportional to *r*^−2^. This indicates that the Alfvén speed *V*_A_ ∝ |*B*|/√*N* should be proportional to *r*^−0.5^. Assuming the upstream speed *V*_n_ is not so different among the four spacecraft, the Alfvén Mach number *M*_A_ (=*V*_n_/*V*_A_) likely decreases as the heliospheric radiance distance increases. This suggests that the CME-driven shock had been developed from a small radial distance, such as at Solar Orbiter location (0.68 AU), and it is consistent with Wijsen et al. (2023), suggesting that the present CME merges with the preceding SIR. The possibility that the interaction between the CME and SIR might play a role in the formation of the PMS in the CME sheath, which was consequently observed as the flip signature in this event, will be further investigated in a future study.

### Forbush decrease and ICME composition on 12 March 2022

In this subsection, we specifically focus on the phenomenon called “Forbush Decrease (FD)” (Forbush 1938). A FD is a temporary depression of the GCR count rate caused by the transient of an ICME, particularly because the ICME acts as a shielding object towards the GCR particles. The amplitude, recovery time and shape of FDs reflect the physical features of the propagating ICME. FDs have been typically studied based on neutron monitor count rate data on the Earth, as GCRs are typically converted into neutron by-products when they cross Earth’s atmosphere. Neutron monitor observations have revealed many features of FDs (e.g., Janvier et al. 2021). However, FD data on the Earth are affected by the shielding effect of the terrestrial magnetosphere and atmosphere. In addition, single point observations on the Earth are insufficient to track changes of FDs that are dependent on the structural variations of large-scale ICMEs. As stated by Freiherr von Forstner et al. (2020), FD studies have been performed with multi-spacecraft data and distance dependence of FDs related to the structural changes and decay of ICMEs have been shown (e.g., Witasse et al. 2017; Winslow et al. 2018). Due to the insufficient FD dataset in the inner heliosphere, employing data from missions like Solar Orbiter, Parker Solar Probe, BepiColombo is desired to investigate the features of near-Sun FDs and understand better how FDs are formed, which is a topic not well understood (e.g., Belov et al. 2023; Davies et al. 2020).

**Fig. 9:**
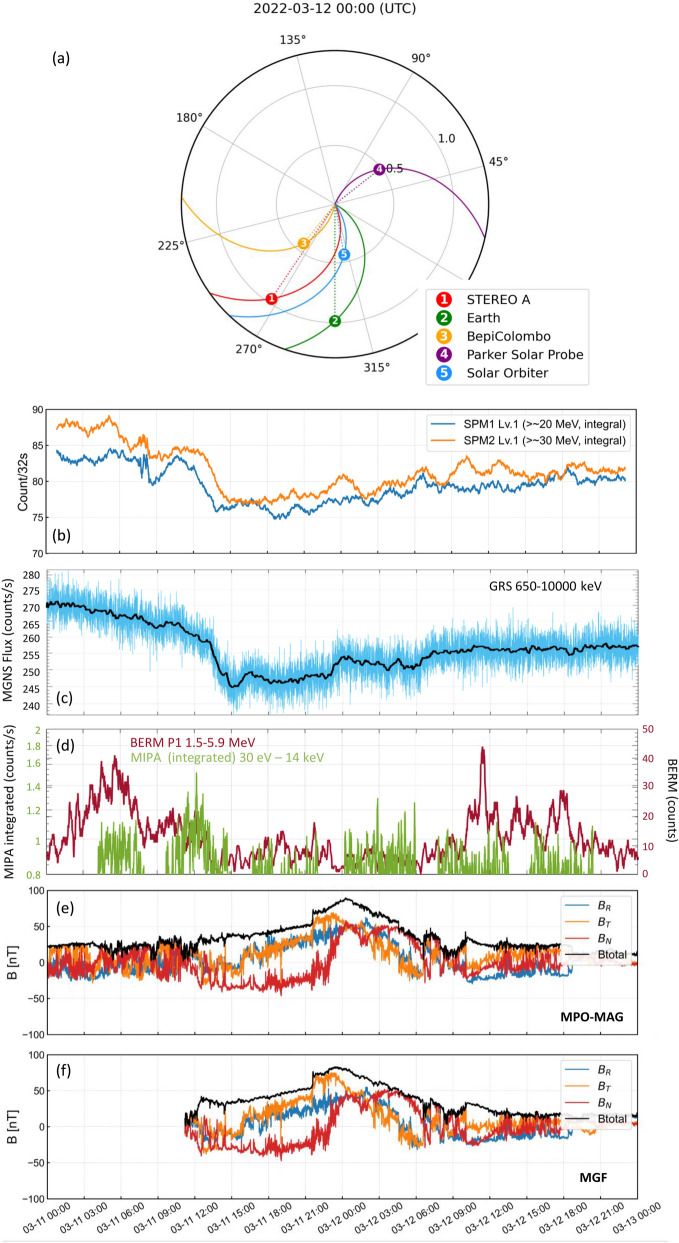
Summary plot of ICME observations acquired by BepiColombo in March 2022. **a** Location of different spacecraft. This panel has been created with the Solar-MACH tool (Gieseler et al. 2022). **b** SPM FD. SPM data corresponds to raw data to which a moving average has been applied with a window of 1600 s. **c** MGNS FD (black line is a moving average of 50 counts window), **d** MIPA and BERM particle (~proton) observations, **e**,**f** MPO-MAG and MGF observations, respectively. The MPO-MAG and MGF data are in RTN coordinates

An ICME hit BepiColombo on 11 March 2022, which was the consequence of a halo CME that erupted from the Sun on 10 March 2022. During that period, several CMEs erupted from the Sun in close temporal proximity, and passed over several spacecraft in the inner heliosphere including BepiColombo, Solar Orbiter, STEREO-A and satellites around Earth. Solar Orbiter and Earth were nearly radially aligned, as well as BepiColombo and STEREO-A along a line about 40° apart in longitude. Both couples of spacecraft were separated by roughly 0.5 AU in radial distance with BepiColombo and Solar Orbiter at about the same distance from the Sun (Fig. 9). In this work, we focus on BepiColombo observations, but we note that this event is currently being studied in conjunction with the other missions as it is ideal to characterise the spatial–temporal evolution of the CME in the radial and azimuthal directions. Moreover, this event has already been analysed by several previous studies from different perspectives such as IPS observations and model comparisons (e.g., Jackson et al. 2023; Laker et al. 2024). In particular, we focus on the FD event detected by SPM and MGNS, the corresponding IMF and low energy particle data acquired by BepiColombo (Fig. 9). MGF (onboard the MIO spacecraft) data have been transported in time to match the MPO-MAG time observations as both instruments are far away from each other on the cruise packed spacecraft configuration. We note that both magnetometer observations are very similar and within the error margins.

**Fig. 10:**
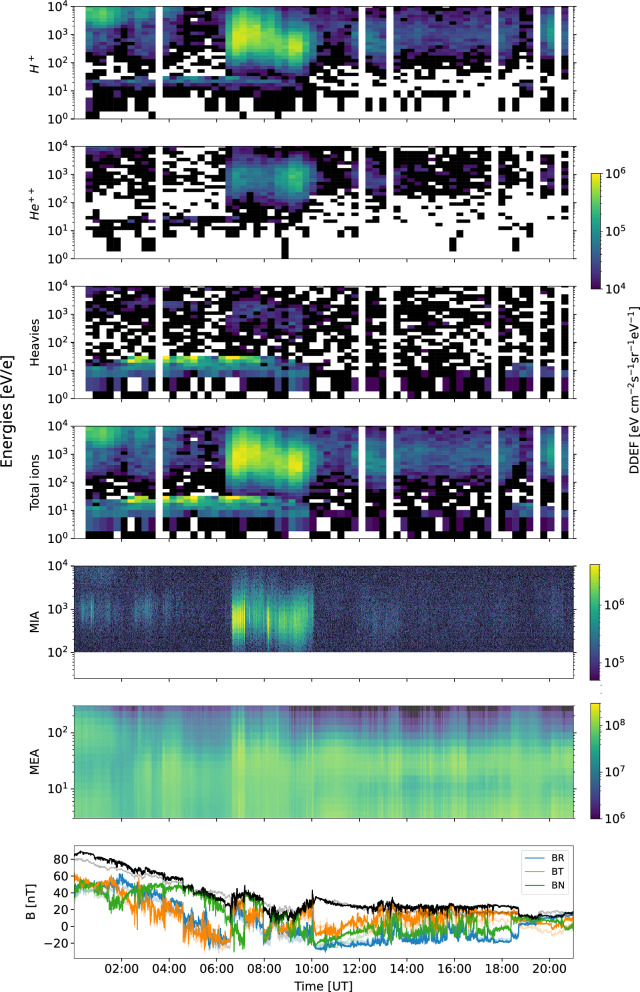
Summary plot of ICME observations acquired by BepiColombo MPPE and MERMAG on 12 March 2022. Four upper panels: MSA observations. Fifth and sixth panels: MIA and MEA energy-time spectrograms of ion and electrons differential fluxes, respectively. Seventh panel: MPO-MAG and MGF magnetic field observations in RTN coordinates.

SPM and MGNS detected a clear FD event on 11 March 10:30 UT, corresponding to the first arrival of the ICME at BepiColombo as seen in the magnetic field observations from both MPO-MAG and MGF, and it was followed by a subsequent rotation of the magnetic field. The MIPA instrument also detected a small proton increase that matches with the arrival of the CME. For consistency, we note that BERM detected a small proton SEP event before the arrival of the CME, but given the time difference between BERM and MIPA peaks, we can conclude that most probably MIPA observations are at lower energies than BERM, and this is why the increase is seen a few hours later in MIPA and not in BERM. Regarding the FD, the decrease phase lasted for ~3 h. It is important to note that the FD started ~1 h later for the SPM channel that is sensitive to higher energies (SPM2, >30 MeV), indicating that each channel is sensitive to a different part of the GCR spectrum. Also note that SPM has been averaged with a moving average with a window of 1600 s to remove noise. The decrease in both channels, particularly the SPM2, coincide pretty well with the observations by MGNS in its gamma ray spectrometer channel that it is sensitive to 650 keV–10 MeV protons. For both instruments, it consists of a ~10% reduction of the GCR flux, and the maximum of the decrease matches the starting time of the ICME magnetic cloud at BepiColombo. After the decrease, there is a slow recovery of the GCR flux that contains some variability, especially in the SPM2 channel and MGNS data, that seems to be related to a couple of magnetic flux ropes, the main one due to the CME around 12 March 00:00 UT and a second one between 12 March 06:00 and 13:00 UT. This second one is better seen in Fig. 10, where a zoom on the CME structure during 12 March is done, and we show the MPPE suite observations. In particular, we show ions measured by MSA and MIA, electrons measured by MEA, and magnetic field observations for reference. As can be seen, the flux rope contains a large quantity of alpha particles and protons. The ions visible at energies below 50 eV are produced by outgassing of water from the spacecraft (Fränz et al. 2024). We do not have MPPE observations the day before but there is evidence for high levels of protons and presence of heavy ions within the magnetic cloud at the beginning of the figure. Electron fluxes are consistent through the entire day, being more intense during the magnetic cloud transit (beginning of the figure) and gradually decreasing through the day. The electron flux is not necessarily larger during the flux rope on 12 March 06:00 UT. This second flux rope could be the result of a second CME that was ejected in the same direction like the previous one, as there was significant activity from the same active region at the Sun the previous days, and the number of alpha particles seen by MSA could corroborate this hypothesis.

This figure shows a nice example of concatenation of CMEs and the different effects they have on the formation of FDs. Future work will include the comparison of these observations to other assets such as Solar Orbiter and Earth in order to evaluate the formation of the FD with radial and angular separations for the same events.

### ICME and a series of SEP events detected between 28 March and 3 April 2022

**Fig. 11:**
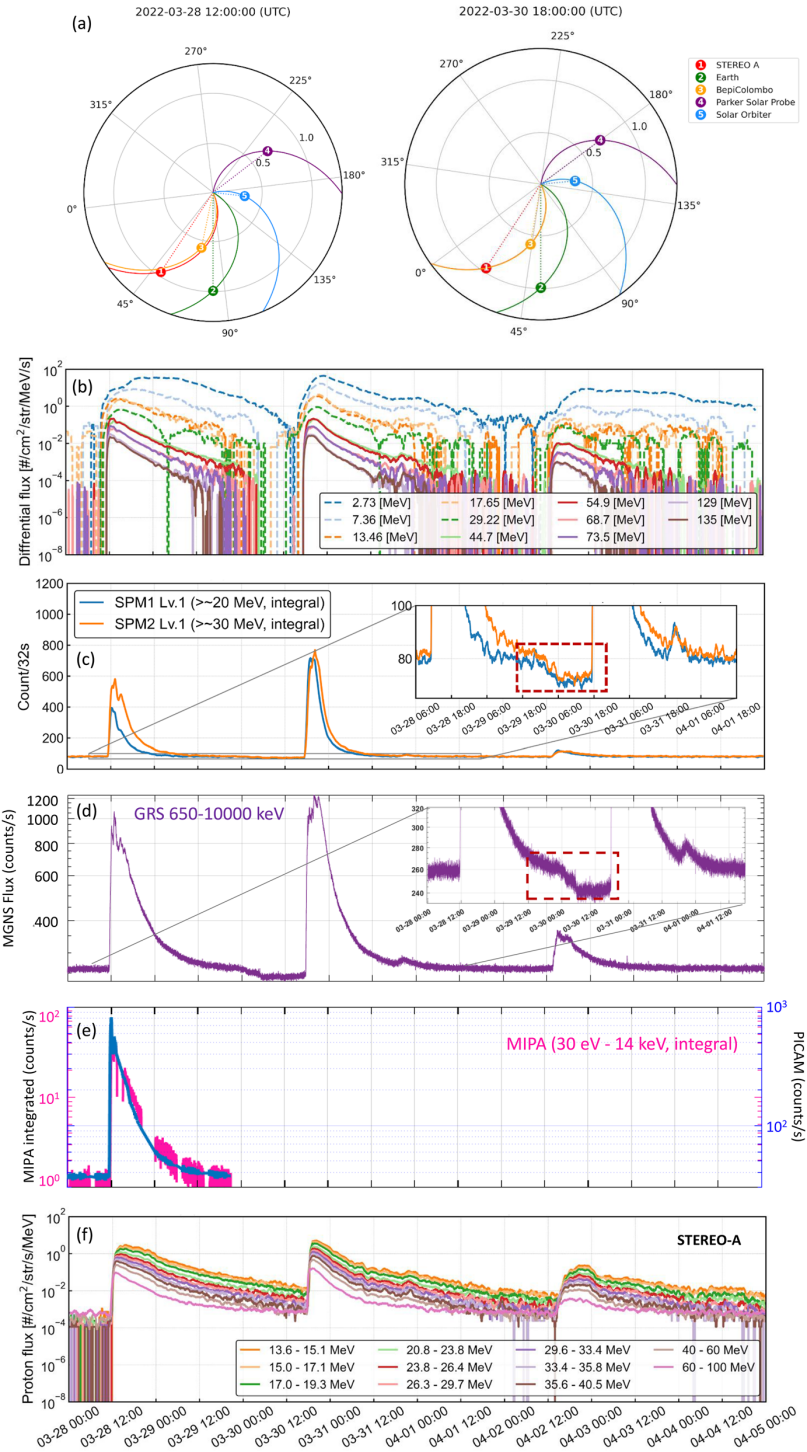
**a** Locations of spacecraft and Earth and their nominal magnetic connections to the Sun on 28 and 30 March 2022. This panel has been created with the Solar-MACH tool (Gieseler et al. 2022). SEP events from 28 March until 5 April 2022 detected by **b** BERM and SPM, **c** SPM only, including a zoom-in on the FD, **d** MGNS, including a zoom-in on the FD **e** MIPA and PICAM in their background counts. **f** STEREO-A HET observations of the SEP

BepiColombo observed a series of SEP events during the period 28 March–5 April 2022. Several spacecraft witnessed these events at multiple locations (Fig. 11). In particular, BepiColombo and STEREO-A were located on the same Parker spiral field line about 0.4 AU apart in the radial direction as shown in Fig. 11a, so we expect that this SEP event was continuously observed on the path. Therefore, this is an ideal event to approach the acceleration process of the SEP in the inner heliosphere by comparing each data. MPO/BERM, MMO/SPM, MGNS and STEREO-A/HET (Von Rosenvinge et al. 2008) cover protons with an energy range of about 10–100 MeV, making them suitable for direct comparison (Fig. 11). Comparing the arrival times of the first SEPs in channels with similar detection energy ranges measured by BERM and STEREO/HET (BERM: 13.46 MeV, HET: 13.6–15.1 MeV), the SEPs reached BERM at 11:45 UT on 28 March and HET at 12:57 UT on 28 March, a difference of about 72 min. The arrival times have been computed based on a threshold value to differentiate between the background and the SEP onset when the latest exceeds this threshold value. The comparison of the shape of the energy spectra is detailed in Fig. 6 of Kinoshita et al. (2025). Their shapes are very similar, suggesting that both probes are observing the same SEP population. We also have background observations from other instruments not designed to measure SEPs, such as PICAM and MIPA, which we present here for comparison. Although not designed for this task, the event on 28 March 2022 was intense enough for protons to penetrate the housing of both instruments, showing a similar response, which indicates that they are both sensitive to the same energetic particles. A full calibration analysis will be done in the near future.

After the first of the SEP events, SPM and MGNS observed a clear FD between 29 and 30 of March 2022 (Fig. 11c, d), coinciding with the arrival time of an ICME (Fig. 11). For both instruments, the decrease was of similar magnitude and was observed simultaneously, being SPM2 closer to MGNS as it is sensitive to higher energies. Since the second SEP event arrived during the recovery phase of the FD (right after the ICME passed by), the recovery time is unclear. However, we can confirm that based on the decrease itself, the amplitude of the FD can be determined as ~10%, similar to the previous one detected on 12 March 2022 (see Sect. 5.3).

For a detailed multi-spacecraft study of this event, please, refer to Stumpo et al. (2025).

### SEP events detected through spacecraft housekeeping data

BepiColombo’s payload is fully designed to measure a wide range of particle energies. However, when these instruments are switched off due to operational constraints, we still can get an indication of SEP passage at BepiColombo. To that end, we have started characterising the response of the spacecraft to SEP events during the cruise journey, mainly using housekeeping sensors that are distributed across the body of the spacecraft (Pinto et al. 2025). In particular, we have used the Error Detection and Correction (EDAC) memory counters (Shirvani et al. 2000). These EDAC counters are pieces of code that protect memories in a spacecraft computer from bit-flips caused by single event upsets (SEU). Every time when energetic particles hit these memories, new errors are accumulated. These memories are typically used to protect the spacecraft and count for the radiation encountered. However, a few previous studies have shown that it can also be used for science purposes. Knutsen et al. (2021) and Rimbot et al. (2024) showed that a long-time series of EDAC errors can be used to characterise the GCR flux, which are especially useful for those missions covering long distances, such as the cruise phase of planetary missions like Rosetta, BepiColombo or JUICE. Moreover, Sánchez-Cano et al. (2023) also showed that the same dataset, when used in short-periods of time, is sensitive to the detection of SEP events, and even more, some specific EDAC datasets, such as those from Mars Express, have been proven to be sensitive to FDs, which are the result of the transit of ICMEs (Viet et al., 2024).

**Fig. 12:**
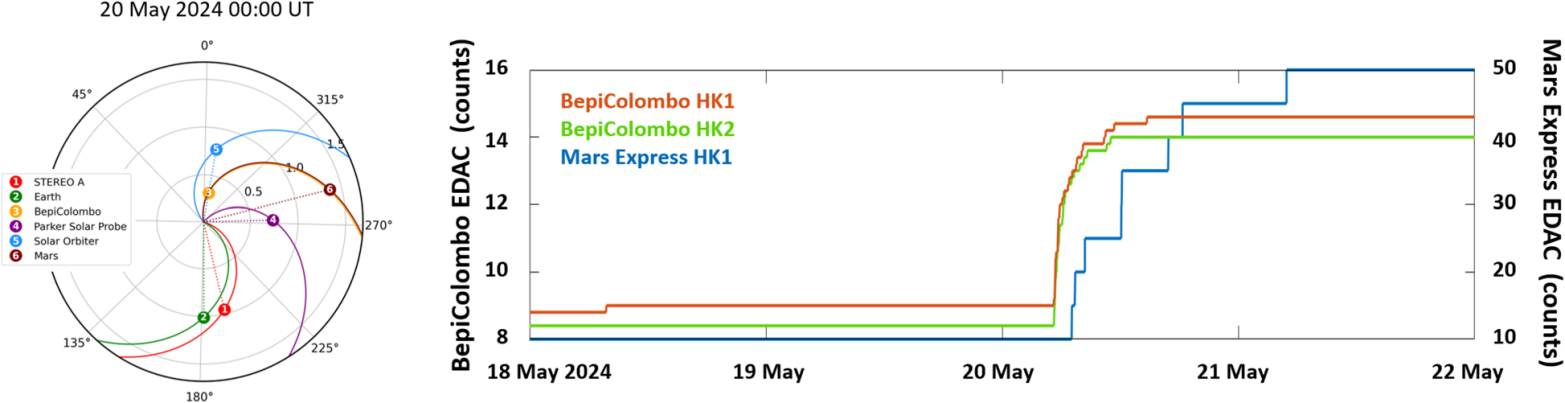
Left: location of different spacecraft on 20 May 2022. This panel has been created with the Solar-MACH tool (Gieseler et al. 2022). Right: EDAC Housekeeping data from BepiColombo and Mars Express. BepiColombo HK1 ID = NCDT2490, BepiColombo HK2 ID = NCDT24A0, Mars Express ID = NACP1300.

In the case of the BepiColombo MPO module, Sánchez-Cano et al. (2023) showed that a very large SEP event that occurred on 15 February 2022 triggered a response of the EDAC memories of the spacecraft. However, no other SEP events have been able to trigger a similar response until 20 May 2024, when the potentially largest SEP event of the cruise hit BepiColombo. We say “potentially” as during this event, all mission payloads were switched off because the European Space Agency was investigating a power anomaly that occurred in the transfer module.^2^ Despite all payloads being disconnected, the EDAC memories onboard BepiColombo detected a clear response at the expected arrival time of this event. Figure 12 shows these observations together with the location of BepiColombo and other missions in the inner heliosphere. As can be seen, a sudden and very significant increase in the EDAC counters was detected at 05:15 UT on 20 May 2024 until 14:37 on the same day. This is a rate of 3.34 errors per hour during the event, when the normal rate is less than an error per day, and this is by far the largest event detected with housekeeping observations so far by this mission, and although it was quite unfortunate that the rest of the scientific payload was not in operation, we can still infer useful information by understanding the response of the EDAC memories to high energetic particles. This event was also seen at other locations, such as at Mars, which was very well magnetically connected to BepiColombo. Figure 12 also shows Mars Express EDAC observations, where the same SEP event also triggered a detection. In this case, it started at 07:00 on 20 May 2024 and lasted until 18:00 on the same day, indicating a rate of 0.72 counts/h. It is not possible to compare the rate of error increase between both spacecraft as it depends not only on the SEP energies, but also on the amount and type of material surrounding the EDAC memories, as well as their locations, which vary from spacecraft to spacecraft and on the memory type and specific sensitivity (Sánchez-Cano et al. 2023). However, we can affirm that in both cases, increases are statistically significant and can be considered real detections of the arrival time of the largest fluxes within the SEP event. Finally, since only two events have triggered a detection in the EDAC memories of BepiColombo, and the event of 20 May 2024 is by far the largest one, we can conclude that despite not having other observations available at BepiColombo, this event was most probably the most intense at higher energies encountered by BepiColombo during the whole trip.

## Discussion

Space Weather, both in the interplanetary medium and for planets, is driven by the Sun’s activity, particularly through large eruptions of plasma that can take many forms, such as CMEs, SIR, high-speed solar wind streams or SEPs. This is an emerging topic, whose real-time forecast is currently very challenging because, among other factors, it needs a continuous coverage of its variability within the whole heliosphere as well as of the Sun’s activity. Understanding how the solar wind and solar transient structures evolve with distance help us to assess how they interact and dissipate their energy with different magnetised/un-magnetised environments, being a key point for possible habitability of planets, moons and even exoplanets.

Solar transients such as CMEs, SIRs, solar flares, solar radio emissions or SEPs can cause extreme and sudden variability in the data observed by any spacecraft encountered by them. Although these are common features in the solar wind, each of them has near unique properties (energy, velocity, etc.) that makes its forecast very complex. Large efforts are being made by both the heliophysics and planetary communities in order to model their propagation, as well as anticipate their strong effects on different plasma–atmospheric systems. Therefore, the long cruise of BepiColombo constitutes an exceptional opportunity for studying the evolution of different solar structures within a half-astronomical unit (AU) of the Sun, particularly in combination with other missions travelling similar distances, such as Solar Orbiter or Parker Solar Probe.

In this work, we have shown the importance of coordinated observations, and how BepiColombo is contributing to a better understanding of the heliosphere and Space Weather. Although this opportunity was not originally planned, it has significantly increased the scientific return of the mission. The availability of complementary in situ and remote sensing instruments onboard various spacecraft missions, combined with ground-based observations and numerical simulations, provides a unique opportunity for unprecedented multi-point measurements of the solar wind plasma and the Sun. This comprehensive observational capability allows the scientific community to investigate a range of fundamental physical processes in the solar wind and transient events including the generation, acceleration, and transport of SEPs, and the characterisation of large-scale heliospheric structures such as ICMEs. The coordinated efforts offer a completer and more dynamic picture of solar phenomena which is crucial for advancing our understanding of the complex interactions within the solar wind and their impacts on Space Weather. Moreover, science during the cruise phase has facilitated a good opportunity for calibrations of the instruments, including cross-calibration between the BepiColombo payloads, but also with other missions when good conjunction opportunities occurred.

Lessons learned during the BepiColombo cruise phase can be applied for other planetary missions such as the ESA JUICE, the JAXA Mars Moons Explorer (MMX), or NASA’s Europa Clipper. Cruise observations are especially important for instrument calibration both on the same missions and with relation to other missions. Planetary flybys, most specifically of Earth where the environment is relatively well known, are a great opportunity to evaluate the response of different instruments and prepare for the science phases of the mission. The same is true for transient events in the interplanetary medium such as ICMEs and SEPs. Since these cannot be predicted, instruments should be operating in science mode as often as possible. When this is not possible, subsets of complementary instruments can also be used for the same purpose.

Additionally, cruise phase measurements can be combined with additional missions for cross-calibration as demonstrated by Khoo et al. (2024), and for scientific analysis as shown in this work. When there is limited observation availability, a detailed observation plan needs to be prepared in advance. For cross-calibration, priority should be given to periods when two spacecraft are magnetically connected in the case of particle detectors, and in the same line of sight in the case of neutral particles. For scientific analysis, many possibilities exist, as demonstrated by Hadid et al. (2021). They presented a full plan of coordinated observation opportunities during the cruise phase of BepiColombo (excluding planetary flybys) to obtain bonus science, perform instrument calibrations, and plan specific observation campaigns during the cruise. This approach, as demonstrated in this work, was very successful.

Finally, we note that due to the stack configuration of the BepiColombo modules during the cruise phase, not all the instruments can be operational all the time, and many of them have strong field-of-view restrictions. For instance, BepiColombo can only measure the solar wind moments during a very limited number of opportunities with the PICAM instrument. This is rather unfortunate, and although the mission can still clearly contribute to heliophysics with those instruments that can be operative, it is a lesson learnt for future missions to plan in advance the cruise science strategy to avoid (if possible) field-of-view obstructions and other operative constraints.

## Conclusions

The cruise phase of BepiColombo has demonstrated to be an important asset for heliophysics science as well as for planetary Space Weather predictions. BepiColombo has been able to outstandingly contribute to characterise the solar wind and transient events encountered by the spacecraft, planetary environments during the flybys of Earth, Venus, and Mercury, and the space radiation environment in the inner Solar System and its evolution with solar activity. We have presented an overview of the cruise observations during 6 years of IMF and solar particle observations, covering half-solar cycle and in situ heliocentric distances between 1.2 and 0.3 AU and remote distances between 11–13 solar radii and 1 AU. Moreover, we have highlighted the most relevant science cases, with the goal of showing the importance of planetary missions to contribute to multi-point observations through the Solar System, especially thanks to the long distances covered by the mission, as well as its particular trajectory within the inner heliosphere. This demonstrates that not only is the calibration of instruments during the cruise important, but it also provides a good opportunity to significantly increase the scientific return of a mission. Lessons learnt should be applied to other missions that are currently starting their cruises (or soon will start), such as JUICE, MMX, ESCAPADE or Europa Clipper, and also for other future missions currently being planned.

## Data Availability

All BepiColombo datasets are publicly available at the European Space Agency Planetary Science Archive, or will be soon after the corresponding proprietary period. Meanwhile, data can be requested to the corresponding authors on reasonable request.

## Footnotes

1 https://www.esa.int/Science_Exploration/Space_Science/BepiColombo/Fourth_Mercury_flyby_begins_BepiColombo_s_new_trajectory.

2 https://www.esa.int/Science_Exploration/Space_Science/BepiColombo/Fourth_Mercury_flyby_begins_BepiColombo_s_new_trajectory.
